# Mendelian randomization based on genome-wide association studies and expression quantitative trait loci, predicting gene targets for the complexity of osteoarthritis as well as the clinical prognosis of the condition

**DOI:** 10.3389/fmed.2024.1409439

**Published:** 2024-06-26

**Authors:** Yiqun Yan, Junyan He, Zelin Xu, Chen Wang, Zhongyao Hu, Chun Zhang, Wendan Cheng

**Affiliations:** ^1^Department of Orthopaedics, The Second Affiliated Hospital of Anhui Medical University, Hefei, China; ^2^Research Center for Translational Medicine, Institute of Orthopaedics, The Second Affiliated Hospital of Anhui Medical University, Hefei, China

**Keywords:** Mendelian randomization, eQTL, osteoarthritis, WGCNA, RF, LASSO, SVM-RFE

## Abstract

**Background:**

Osteoarthritis (OA) entails a prevalent chronic ailment, marked by the widespread involvement of entire joints. Prolonged low-grade synovial inflammation serves as the key instigator for a cascade of pathological alterations in the joints.

**Objective:**

The study seeks to explore potential therapeutic targets for OA and investigate the associated mechanistic pathways.

**Methods:**

Summary-level data for OA were downloaded from the genome-wide association studies (GWAS) database, expression quantitative trait loci (eQTL) data were acquired from the eQTLGen consortium, and synovial chip data for OA were obtained from the GEO database. Following the integration of data and subsequent Mendelian randomization analysis, differential analysis, and weighted gene co-expression network analysis (WGCNA) analysis, core genes that exhibit a significant causal relationship with OA traits were pinpointed. Subsequently, by employing three machine learning algorithms, additional identification of gene targets for the complexity of OA was achieved. Additionally, corresponding ROC curves and nomogram models were established for the assessment of clinical prognosis in patients. Finally, western blotting analysis and ELISA methodology were employed for the initial validation of marker genes and their linked pathways.

**Results:**

Twenty-two core genes with a significant causal relationship to OA traits were obtained. Through the application of distinct machine learning algorithms, MAT2A and RBM6 emerged as diagnostic marker genes. ROC curves and nomogram models were utilized for evaluating both the effectiveness of the two identified marker genes associated with OA in diagnosis. MAT2A governs the synthesis of SAM within synovial cells, thereby thwarting synovial fibrosis induced by the TGF-β1-activated Smad3/4 signaling pathway.

**Conclusion:**

The first evidence that MAT2A and RBM6 serve as robust diagnostic for OA is presented in this study. MAT2A, through its involvement in regulating the synthesis of SAM, inhibits the activation of the TGF-β1-induced Smad3/4 signaling pathway, thereby effectively averting the possibility of synovial fibrosis. Concurrently, the development of a prognostic risk model facilitates early OA diagnosis, functional recovery evaluation, and offers direction for further therapy.

## 1 Introduction

Osteoarthritis (OA), being the most widespread and commonly encountered chronic joint ailment among the aging population worldwide, involves not solely alterations in a singular tissue but the entirety of the joint, primarily encompassing progressive degradation of articular cartilage, sustained low-grade inflammation of the synovium, injury and reconstruction of subchondral bone, and the formation of osteophytes within the joint space, among other aspects (1, 2). Among these, synovitis emerges as the pivotal factor contributing to this array of pathological alterations. Numerous etiological factors contribute to the onset of OA, encompassing elements such as age, gender, genetic predisposition, and body weight (3). However, due to the absence of a clearly delineated pathogenic mechanism, there currently exists no method capable of curing or ameliorating the condition of OA. In the early stages of the disease, relief from pain is often sought through the application of exercise therapy and nonsteroidal anti-inflammatory drugs (NSAIDs). However, in the advanced stages, joint replacement surgery stands as the sole pathway for OA patients to achieve a complete restoration of joint function and attain a curative outcome. This imposes significant economic burdens on the healthcare system and the households of affected individuals (4). However, it is regrettable that we must acknowledge OA as a silent malady, occurring before the manifestation of typical clinical symptoms and radiographic changes. During this protracted subclinical phase, irreversible damage and alterations to articular cartilage may have already occurred (5, 6). Therefore, the exploration of highly sensitive and efficient diagnostic biomarkers, along with the investigation of their associated mechanistic pathways, holds great allure for the diagnosis and treatment of OA.

Mendelian randomization (MR) has ascended to prominence as a dependable methodology for investigating potential causal relationships. At its core, this methodology employs single nucleotide polymorphisms (SNPs) as instrumental variables (IV) to infer the causal relationships between exposure factors and study outcomes (7, 8). In this endeavor, we used summary-level data from genome-wide association studies (GWAS) on OA with expression quantitative trait loci (eQTL) data to explore genes associated with expression levels and complex traits. Subsequently, in conjunction with the GEO database, differential analysis and weighted gene co-expression network analysis (WGCNA) analysis were conducted on the corresponding dataset samples. This process identified core genes that demonstrate a significant causal relationship with OA traits. Building upon this foundation, the application of three machine learning algorithms was integrated, ultimately confirming two genes (RBM6 and MAT2A) as gene targets for the complexity of OA. Simultaneously, corresponding ROC curves and nomogram models were established for the assessment of clinical prognosis in patients. Finally, we preliminarily demonstrated that MAT2A effectively mitigates the potential for synovial fibrosis by participating in the regulation of S-adenosylmethionine (SAM) synthesis, thereby inhibiting the activation of the TGF-β1-induced Smad3/4 signaling pathway.

## 2 Materials and methods

### 2.1 Data source and preprocessing

Retrieve eQTL data from the eQTLGen consortium, encompassing 31,684 samples of blood and peripheral blood mononuclear cells, spanning 19,942 genes (9). Utilize this dataset as the exposure data and filter cis-eQTLs with *p*-value<5 × 10^−8^ for subsequent bidirectional MR analysis. During this process, establish the cis-regulatory region within a range of 10,000 kilobases on both flanking sides of the coding sequence, and perform linkage disequilibrium clustering with *R*^2^ < 0.001. Simultaneously, only SNPs with effective allele frequency (EAF) >0.01 and *F*-statistic >10 were retained, while corresponding palindromic SNPs were removed to avoid confounding and weak instrumental variable bias.

Retrieve the osteoarthritis dataset (ebi-a-GCST007090) from the GWAS database, which consists of 403,124 samples, including 24,955 cases in the afflicted group and 378,169 controls in the reference group.

Acquire human osteoarthritis microarray datasets from the GEO database, encompassing GSE55235-GPL96, GSE55457-GPL96, GSE82107-GPL570, GSE1919-GPL91, GSE12021-GPL96, and GSE206848-GPL570. Among these, the first three datasets serve as training set data, totaling 57 samples, including 27 health control samples and 30 OA samples. The latter three datasets function as validation set data, comprising an overall sample of 43 samples, including 21 health control and 22 OA cases. Eliminate batch effects between datasets from different platforms utilizing the “limma” and “SVA” packages in R software, standardize and merge the samples, and visualize the results using PCA functionality (10, 11).

### 2.2 Mendelian randomisation analysis

This analysis was conducted entirely using R (version 4.3.2). No new data were collected in the process; instead, publicly available GWAS statistical summaries were utilized. Hence, no additional ethical approval was required. We first identified genetic variants (SNPs) that are significantly associated with the exposure of interest (gene expression levels) from GWAS data. Select these SNPs (*p*-value <5 × 10^−8^) as IVs to explore causal effects between exposure data and outcome data (12). The eligible IVs must satisfy the following three conditions: (1) correlation assumption: SNPs exhibit close associations with the exposure (eQTL data). (2) Independence assumption: IVs are unrelated to any unmeasured confounders of OA. (3) Exclusion assumption: IVs are only associated with OA through eQTL and not through other pathways (i.e., they have no direct association with the outcome). During this process, establish the cis-regulatory region within a range of 10,000 kilobases on both flanking sides of the coding sequence, and perform linkage disequilibrium clustering with *R*^2^ < 0.001. Simultaneously, only SNPs with effective allele frequency (EAF) >0.01 and *F*-statistic >10 were retained, while corresponding palindromic SNPs were removed to avoid confounding and weak instrumental variable bias. Subsequently, harmonize the instrumental variables (IVs) with the outcome data. Assessed through five methods, namely MR Egger, inverse variance weighted (IVW), weighted median, weighted mode and simple mode, utilizing the “TwoSampleMR” package within the R software (13). Filter the corresponding results according to the following conditions: (1) *p* < 0.05 for the IVW method. (2) Consistency in the direction of the odds ratio (OR) among the five methods, where all OR values are either >1 or <1. (3) *p* > 0.05 for heterogeneity. Finally, the Wald ratio method was used to estimate the effect of each SNP. Cochrane’s *Q* test assessed heterogeneity between SNP instruments. Sensitivity analysis methods included MR Egger regression test and leave-one-out analysis, with the former assessing multiplicity and the latter revealing the contribution of individual SNPs to significant results. Based on the MR results, we prioritized genes with strong evidence of a causal relationship with OA.

### 2.3 Identifying differentially expressed genes and performing weighted gene co-expression network analysis

Utilizing the “limma” package to identify differentially expressed genes (DEGs) between samples from HC and those with OA. The criteria are set to *p* < 0.05 and |logFC| >0.585 for standardization. Visualization of the outcomes is performed using the “pheatmap” function.

Employing the “WGCNA” package to build a gene co-expression network based on the expression profiles of samples from OA and HC groups (14). Choosing a gene subset with a standard deviation exceeding 0.5 for further analysis. Utilizing the “goodSampleGenes” function to ensure the absence of missing values or anomalies in the dataset. Employing the “pickSoftThreshold” function to determine the optimal soft threshold. Upon this foundation, transforming the data matrix of gene expressions into the corresponding adjacency matrix. Subsequently, identifying gene modules through a topological overlap clustering method. By computing module eigengenes (ME) and merging between similar modules, a hierarchical clustering dendrogram is then generated. Integrating pertinent phenotypic data to evaluate gene significance (GS) and module significance (MS). Thereby, elucidating the significance between associated genes and clinical information, as well as the correlation between modules and models.

### 2.4 Acquisition of core genes

Initially, we filter the outcomes of Mendelian randomization. The filtration criteria are as follows: (1) *p* < 0.05 for the IVW method. (2) Consistency in the direction of the odds ratio (OR) among the five methods, where all OR values are either >1 or <1. (3) *p* > 0.05 for heterogeneity. Subsequently, we select modules from the WGCNA results that demonstrate associations with the disease and possess a *p*-value <0.05, designating these as core modules. Finally, we make use of the “VennDiagram” package to intersect the obtained differentially expressed genes, genes included in the core modules from WGCNA, and genes resulting from Mendelian randomization (15). The genes obtained from this intersection are designated as core genes.

### 2.5 Functional enrichment analysis and chromosomal localization

Utilizing the “clusterProfiler” and “enrichplot” packages to perform Gene Ontology (GO) and Kyoto Encyclopedia of Genes and Genomes (KEGG) functional enrichment analyses on the identified core genes (16). The criteria are established as *p* < 0.05 and *q* < 0.05 for standardization. Gene Ontology (GO) terms include Biological Processes (BP), Molecular Functions (MF), and Cellular Components (CC). Display the results based on enrichment ranking thresholds, such as *p*-values. Utilize the “circlize” package for visualizing the chromosomal localization of core genes.

### 2.6 Analysis of immune cell infiltration and correlation

Employing the CIBERSORT algorithm to assess the composition and abundance of 22 immune infiltrating cell types in both HC and OA samples. This algorithm operates through deconvolution and is grounded in the principles of linear support vector regression (17). The results are visually represented as a heatmap. Apply the rank-sum test to assess differences in immune cell infiltration between two sample groups, considering *p* < 0.05 as the threshold for indicating significant distinctions. Conducting a thorough investigation of core genes and infiltrating immune cells in the samples using Spearman correlation analysis. Finally, visualizing the results of both analyses using the “ggplot2” functionality.

### 2.7 Selection of disease-specific characteristic genes

Utilizing the “randomForest” package, employ the Random Forest (RF) method for further refinement in the selection of core genes (18). This constitutes a machine learning approach that integrates multiple decision trees, mitigates overfitting, and is adept at handling high-dimensional and imbalanced data. It proves advantageous for the screening and identification of characteristic genes.

Utilizing the “e1071” package, employ the Support Vector Machine Recursive Feature Elimination (SVM-RFE) method for further refinement in the selection of core genes (19). This is a widely employed supervised machine learning method in classification and regression analysis, possessing high discriminatory capabilities for the selection of characteristic genes.

Utilizing the “glmnet” package, employ the Least Absolute Shrinkage and Selection Operator (LASSO) regression method for further refinement in the selection of core genes (20). This is a machine learning method for variable selection of characteristic genes through regularization, demonstrating unique superiority in assessing high-dimensional data.

Cross-referencing the findings obtained from the three algorithms to identify the OA diagnostic marker genes.

### 2.8 Construction of ROC curves and nomograms for the clinical prognosis model

Utilizing the “pROC” package, construct ROC curve models for diagnostic signature genes. Calculate the area under the curve values to preliminarily assess the diagnostic effectiveness of the signature genes for OA (AUC >0.7 is considered indicative of accuracy).

Utilizing the “rms” package, construct a nomogram for the risk prognosis model. Infer the occurrence rate of osteoarthritis based on the total score corresponding to the sum of scores from individual diagnostic signature genes. A higher score indicates a higher likelihood of disease occurrence. The “regplot” package is employed to draw a calibration curve, where a better prediction accuracy is indicated by a slope closer to 1. The “ggDCA” package is employed for drawing a decision curve, with a net benefit value >0 signifying a well-performing predictive model.

### 2.9 Collection of clinical specimens and cell culture

The clinical specimen collection plan for synovial tissues from both HC and OA has received approval from the Ethics Committee of the Second Affiliated Hospital of Anhui Medical University (Approval Number: YX2022-104). The procedures were conducted in accordance with relevant guidelines and regulations. Among these, synovial specimens from HC were obtained from two patients who underwent limb amputation due to accidents. Synovial specimens from patients with severe OA were collected from four patients undergoing surgical treatment. After specimen collection, synovial cells were immediately extracted for primary culture. Notably, the cells derived from OA samples were passaged for no more than five generations. All cells were cultured in DMEM medium supplemented with 10% fetal bovine serum, under the conditions of 37°C and 5% CO_2_ in a humidified incubator. Additionally, we characterized the composition of 4–5 generations of primary synoviocytes using flow cytometry. Following trypsin digestion, cells were washed through centrifugation and resuspended in PBS. Subsequently, cell closure was achieved using a 5% BSA solution. The cells were then incubated with CD45 antibody at 4°C in the absence of light. Following washing steps, flow cytometry was employed to detect the expression of CD45 on the cell surface, with CD45-negative cells identified as synovial fibroblasts. Please refer to Supplementary Figure S1 for further details.

The following is the exact procedure for synoviocyte extraction: after excising the fresh synovial tissue, it was immersed in pre-cooled PBS. Using scissors and forceps, efforts were made to remove as much fat and muscle tissues from both sides of the synovium to obtain a pure, white synovial tissue. Subsequently, the tissue was minced into pieces as small as possible, approximately 2 mm^3^ in size, and placed into a 5 mL EP tube. Then, α-MEM containing 2 mg/mL Collagen I at a volume 2–3 times that of the synovial volume was added. The tube was then placed in a 37°C incubator for digestion for 3–4 h. During this period, tissue digestion was monitored, and the tube was gently shaken every half hour. After digestion, the mixture was filtered through a 70 μm filter and centrifuged at 1,800 rpm for 5 min, followed by discarding the supernatant. The cells at the bottom of the centrifuge tube were resuspended in DMEM, centrifuged for 5 min, and this washing step was repeated twice. Finally, the cells were cultured in culture flasks with DMEM medium containing 10% fetal bovine serum.

### 2.10 Transient transfection technique

The siRNA targeting MAT2A was designed and constructed by Shanghai Gima Pharmaceutical Co., Ltd. (Sequence: 5′-ACACAUUGGAUAUGAUGAUTT-3′). Cultivate synovial cells in a six-well plate and transfect MAT2A using Lipofectamine 2000 (Invitrogen, Carlsbad, CA). Transfect cells and culture for 5 days, then assess the knockout efficiency of the target gene through western blotting analysis.

### 2.11 ELISA method is used to determine the expression of SAM in cells

The SAM reagent kit was purchased from Jiangsu Meimian Industrial Co., Ltd. On the enzyme-linked plate, set up standard wells, blank wells, and wells for the test samples. Add the corresponding reagents to each well. After sealing the plate, it was placed in a sterilized humidified incubator with the following conditions: 37°C, 5% CO2, for 30 min. Subsequently, each well was washed for 30 s, repeated 5 times. Enzyme-linked reagent (50 μL) was added, and the plate was placed back in the humidified incubator. After completion, it was washed again. Color reagent A and B were added in a 1:1 ratio, and the plate was incubated in the dark for 10 min. Termination solution (50 μL) was added to stop the reaction. Using an enzyme reader, the absorbance of SAM was calculated at 450 nm. To determine the concentration of SAM in each sample, and present the results in a bar chart.

### 2.12 Western blotting analyze

The cells were lysed using RIPA lysis buffer containing proteinase inhibitors (Beyotime Biotech #P0013B), and the total protein content was quantified using the BCA assay kit (Thermo #PL212989). Separate proteins using 7.5% SDS-PAGE and transfer them onto a PVDF membrane. Following blocking with 5% skim milk, membranes are incubated with primary antibodies against RBM6 (1:750, AB_2720243), MAT2A (1:1000, AB_2718781), TGF-β1 (1:2500, AB_2202039), SMAD3 (1:2500, AB_2552126), SMAD4 (1:2500, AB_2552158), GAPDH (1:2500, AB_2107311), αSMA (1:500, AB_2792219), and COL1A1 (1:750, AB_2803705) at 4°C overnight. All the mentioned antibodies are rabbit polyclonal antibodies and are sourced from Thermo Fisher, United States. Subsequently, incubate the membrane with secondary antibodies at 37°C for 2 h, and visualize the protein bands using a microplate chemiluminescence system (Share-BIO, SB-WB012). Finally, utilize ImageJ software (version 1.80v) to normalize and analyze the bands of each target using the GAPDH band as a reference.

## 3 Results

### 3.1 Causal effects between gene expression and OA

After correlation analysis and removal of linkage disequilibrium, we obtained a total of 5,430 genes, involving 26,152 strongly correlated SNPs (*F*-value >10, Supplementary Table S1). Using instrumental variables (SNPs) derived from the exposure data, we obtained the summary results of 22,306 SNPs associated with OA from the osteoarthritis GWAS dataset (Supplementary Table S2). There is no evidence indicating heterogeneity among these IVs (Supplementary Table S3) or multiplicity (Supplementary Table S4). Perform Mendelian randomization analysis based on a unified reference allele direction and calculate the odds ratios (OR) for the corresponding results. Subsequently, further screening of the results of Mendelian randomization according to preset criteria resulted in a final selection of 318 genes causally connected to the onset and progression of OA, including 173 low-risk genes and 145 high-risk genes (Supplementary Table S5). High/low-risk genes refer to genes where an increase in gene expression is associated with an increased or decreased likelihood of osteoarthritis occurrence, respectively.

### 3.2 PCA analysis and identification of DEGs

**Figure 1:**
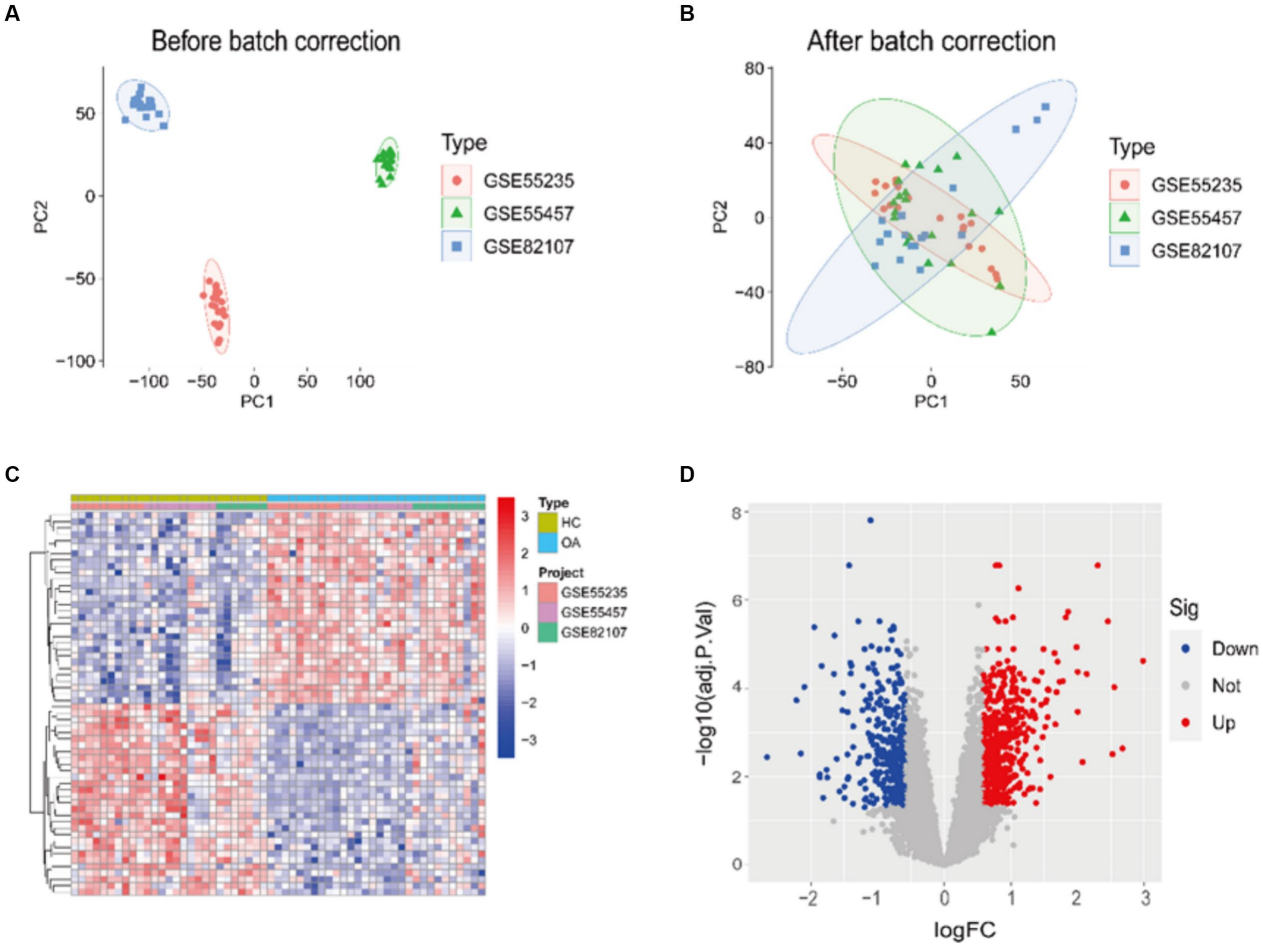
PCA and DEGs identification results. **(A,B)** PCA of samples before/after integration of datasets. **(C,D)** A heatmap and a volcano plot showing how DEGs are expressed in the OA and HC samples.

Prior to batch correction and standardization integration, the PCA results demonstrate no overlap among the three datasets (Figure 1A). However, following integration, the three datasets exhibit overlap (Figure 1B). This suggests that the integrated data can be considered as a unified batch for subsequent analysis. Based on this, we conducted differential analysis between HC and OA samples, revealing significant differences between the two (Figures 1C,D). In total, 2,258 DEGs were identified, comprising 978 genes significantly downregulated and 1,280 genes significantly upregulated.

### 3.3 Identification of key modules using WGCNA

**Figure 2:**
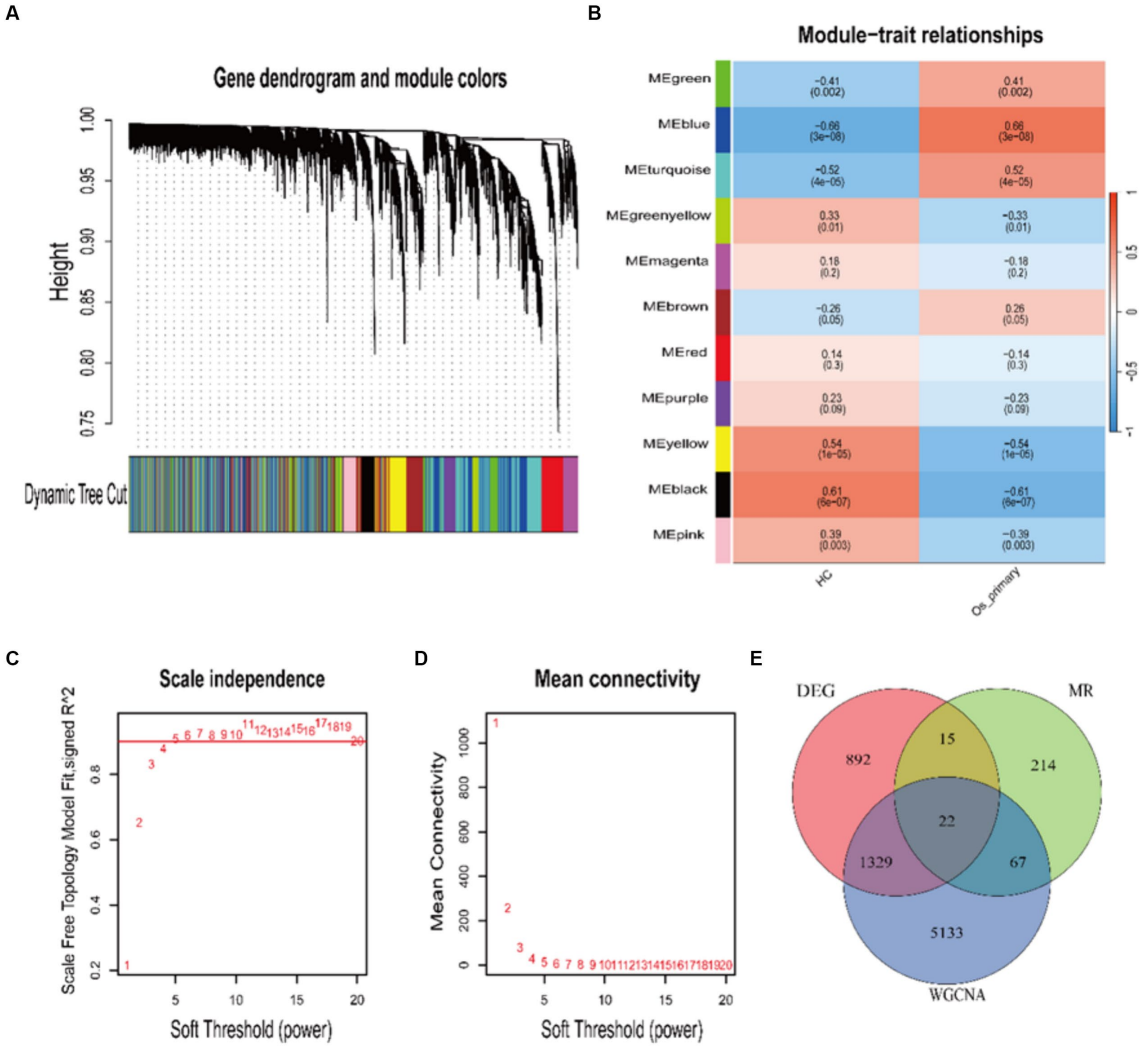
WGCNA Analysis. **(A)** Hierarchical clustering dendrogram. Each branch represents a gene, and the colors below each branch represent co-expressed modules. **(B)** Heatmap depicting the correlation and significance between modules and clinical trait features. **(C,D)** Depiction of the scale-free fitting index and average connectivity for the co-expression networks associated with OA and HC across various soft-thresholding powers. **(E)** Venn diagram intersection of MR genes, DEGs, and WGCNA key module genes.

We explored the relationship between genes and traits by performing WGCNA. Opting for the optimal soft-thresholding power *β* = 5 (scale-free *R*^2^ = 0.9), we established a co-expression network involving HC and OA samples (Figures 2C,D). By employing dynamic hybrid cutting, a total of 11 modules with distinct colors were acquired (Figure 2A). Simultaneously, compute the Pearson correlation coefficients and significance levels for the relationship between each module and clinical trait features. The corresponding results are represented in a heatmap format (Figure 2B). Remarkably, the black, blue, brown, green, yellow-green, pink, tan, and yellow modules are recognized as key modules, comprising 335, 1,729, 1,005, 613, 159, 252, 1,793, and 665 genes, respectively.

### 3.4 Obtaining intersecting core genes

**Figure 3:**
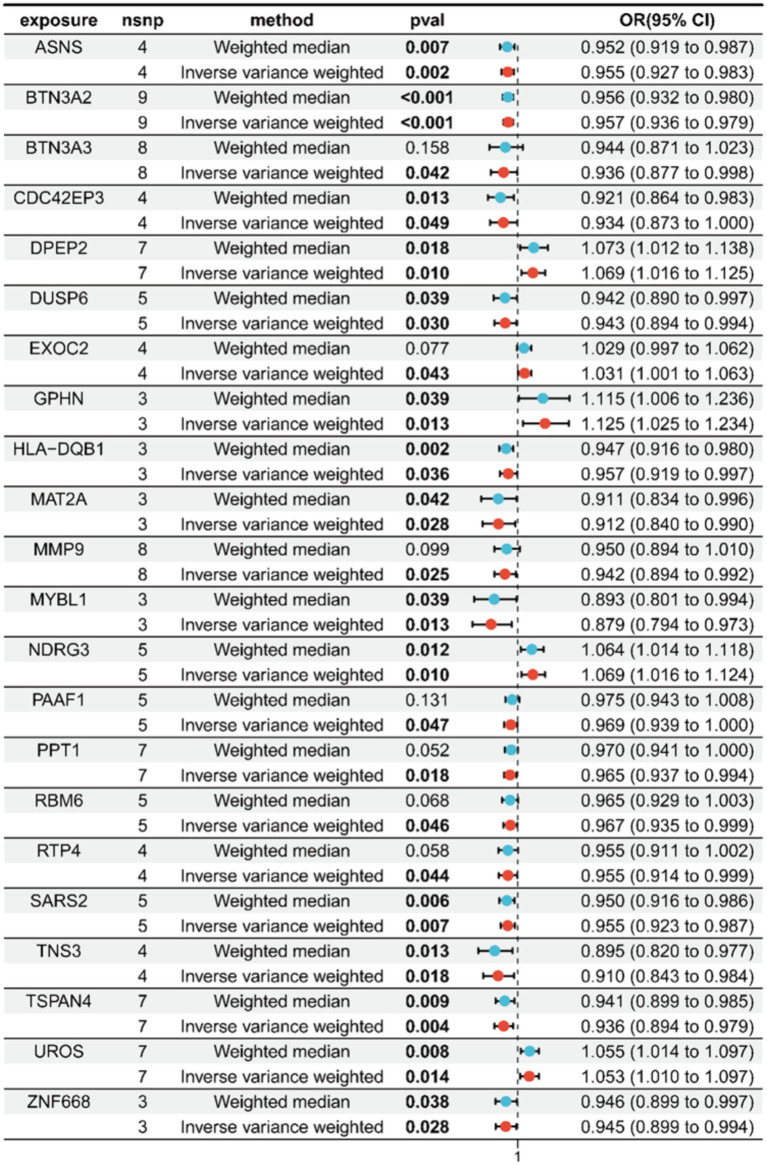
Forest plot of Mendelian randomization for the 22 core genes.

**Figure 4:**
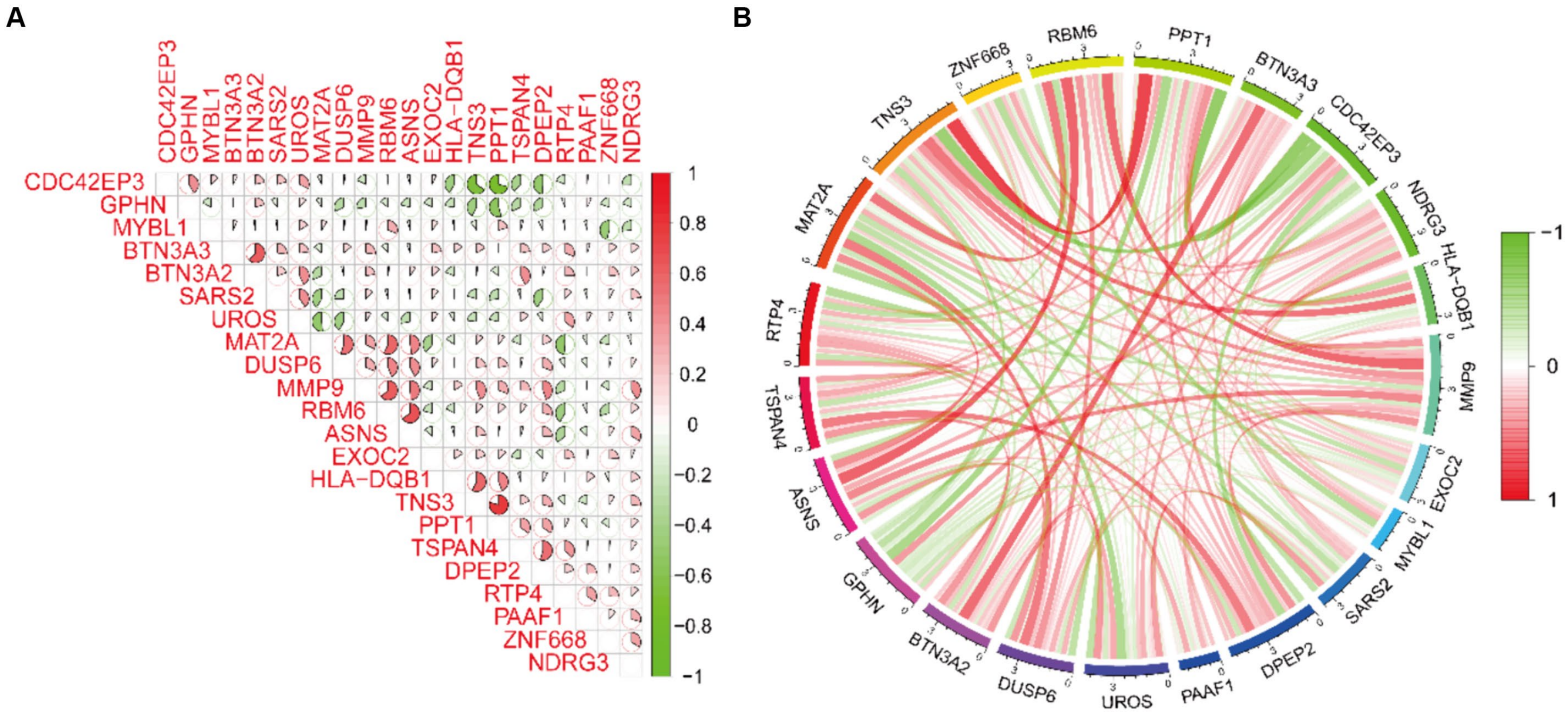
Spearman correlation analysis of core genes. **(A,B)** The correlation heatmap and circos plot demonstrate the functional associations or co-regulation among the 22 core genes.

By cross-referencing the 318 genes causally connected to the onset and progression of OA from the MR results, the 2,258 DEGs, and the genes from the key modules identified by WGCNA, we identified 22 core genes (Figure 2E). Subsequently, we visually represented the Mendelian randomization results of the 22 core genes in the form of a forest plot (Figure 3). Specifically, genes with *p* < 0.05 in the IVW method are regarded as genes causally associated with the outcome factor (OA). Employing the dashed line in the center of the forest plot as a reference, we evaluated genes associated with high and low risk. Genes with OR values to the left of the dashed line are <1, indicating a low risk, while those to the right are >1, indicating a high risk. It can be observed that within the 95% confidence interval, all core genes can be classified into 17 low-risk genes and 5 high-risk genes. Additionally, we conducted an analysis of the correlation among these high/low-risk genes, displaying their functional associations or co-regulation in the form of a heatmap or a circos plot (Figures 4A,B). This can provide insights into the molecular mechanisms driving the disease. Genes that are co-regulated or functionally related may have a higher likelihood of being key players in OA. This might lay the foundation for future research.

### 3.5 Functional enrichment analysis of core genes

**Figure 5:**
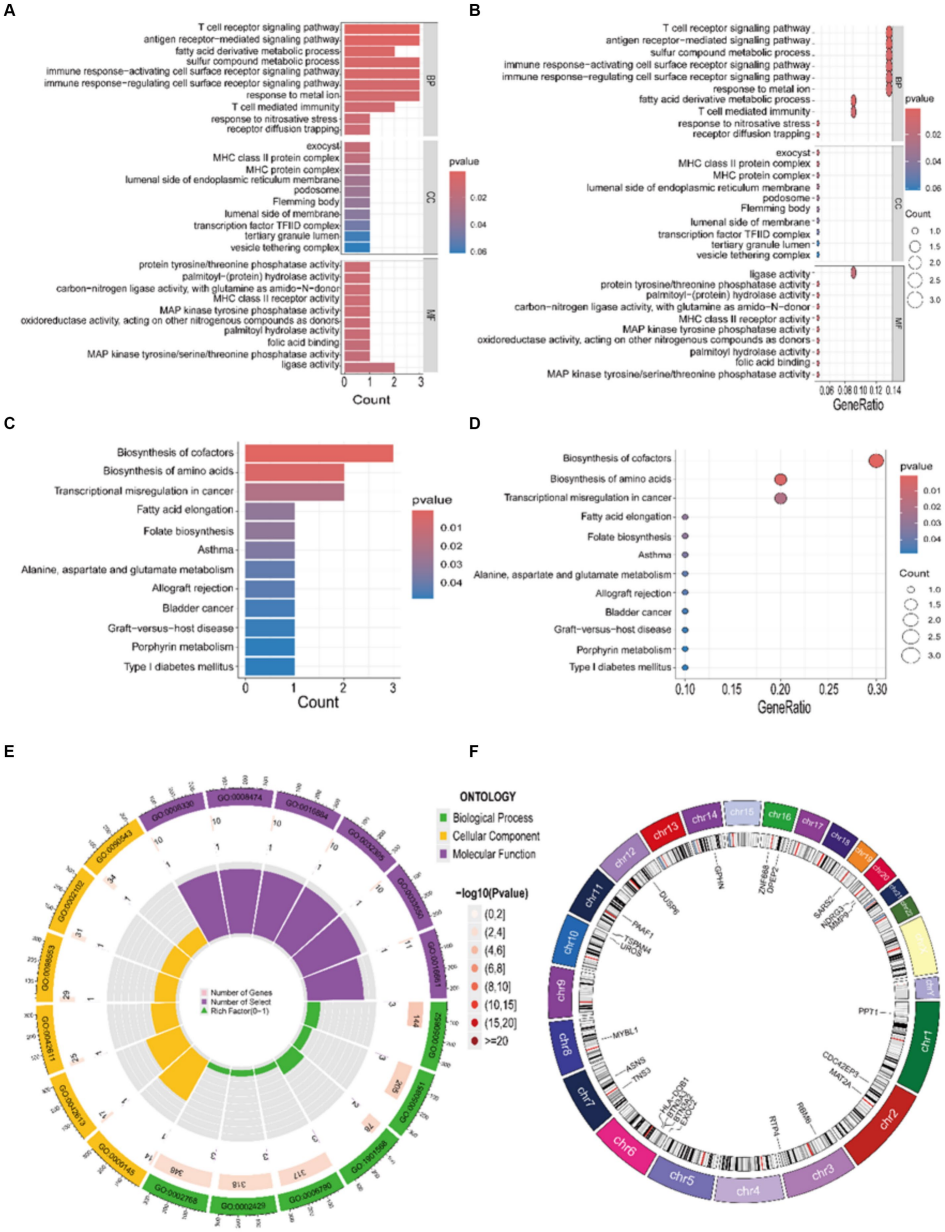
Enrichment results of GO and KEGG. **(A,B,E)** GO bar chart, bubble chart, and circle plot illustrating the top 10 entries determined by the enrichment ranking threshold, such as the *p*-value. **(C,D)** KEGG bar chart and bubble chart illustrating the top 12 entries determined by the enrichment ranking threshold, such as the *p*-value. **(F)** Gene chromosome localization circle plot.

To elucidate the biological functions in which core genes are involved, we conducted GO and KEGG enrichment analyses. The outcomes from the GO enrichment analysis, as depicted in Figures 5A,B,E, unveil: (1) in the BP category, these genes are significantly enriched in processes such as the sulfur compound metabolic process, T cell receptor signaling pathway, sulfur compound biosynthetic process, and T cell-mediated immunity. (2) In terms of CC, there is a potential involvement in the synthesis of substances such as exocyst and MHC class II protein complex. (3) In the MF category, there is a close association with MHC class II receptor activity, ligase activity, and protein tyrosine/threonine phosphatase activity. The results from the KEGG analysis, as depicted in Figures 5C,D, suggest that these genes are primarily involved in pathways related to biosynthesis of amino acids and biosynthesis of cofactors. Furthermore, for an enhanced comprehension of the structure, function, and genetic transmission patterns of the core genes, we constructed a circular plot depicting chromosome localization for the relevant genes (Figure 5F).

### 3.6 Correlation between immune cell infiltration in OA and the co-expression of core genes

**Figure 6:**
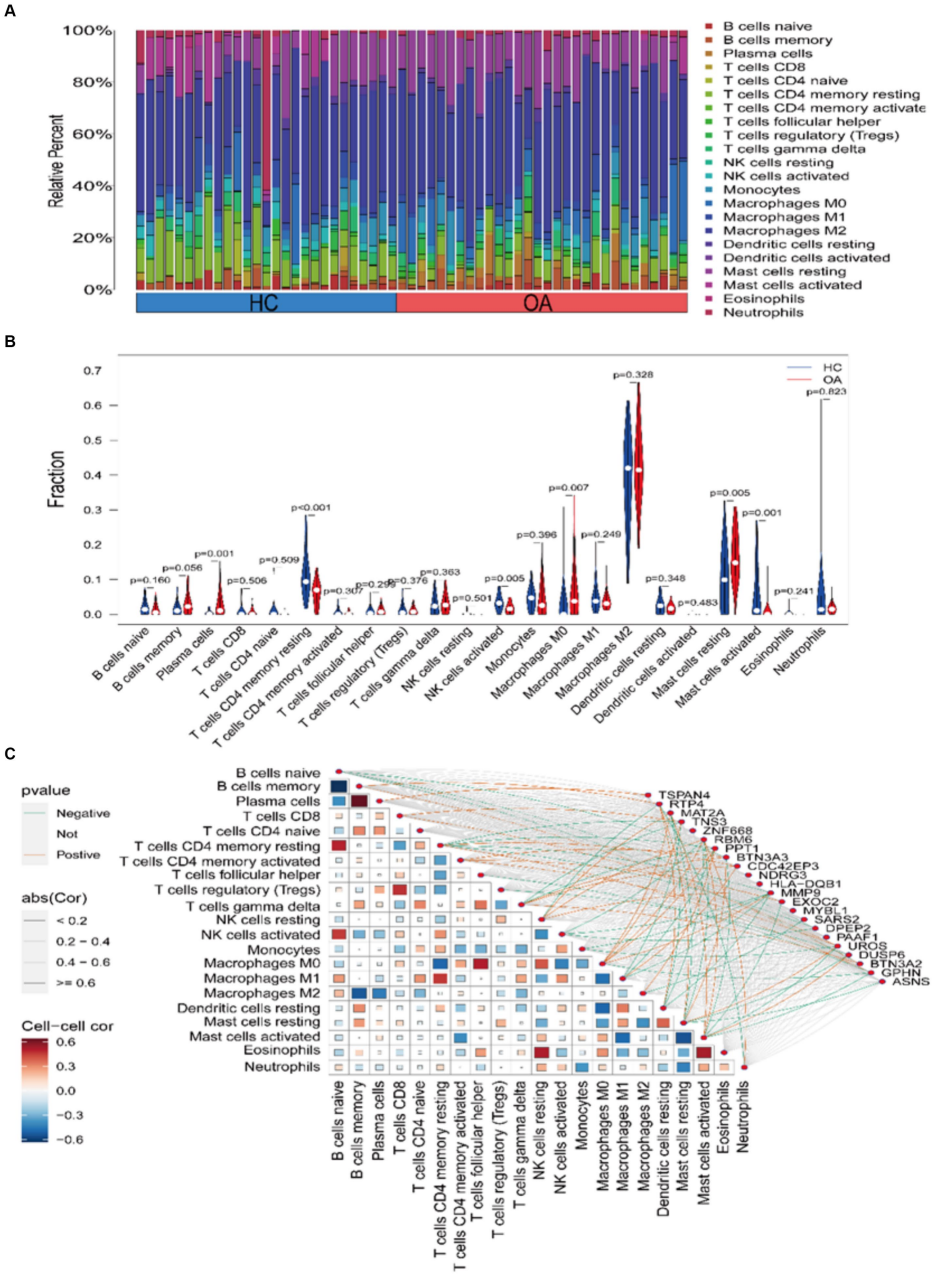
Assessment and visualization of immune cell infiltration. **(A)** Composition and abundance of 22 immune cell types in OA and HC samples. **(B)** Violin plot depicting differential analysis of immune cell infiltration. **(C)** Correlation between core gene expression and immune cell infiltration in the samples.

To examine the correlation between immune cell infiltration in OA and the expression of core genes, we initially employed the CIBERSORT algorithm to assess the composition and abundance of 22 immune cell types in both OA and HC samples, as illustrated in Figure 6A. Expanding on this, a differential analysis of immune cell infiltration between the two sample types was performed, as depicted in Figure 6B. A notable escalation in the infiltration levels of plasma cells, *p* = 0.001, and macrophages M0, *p* = 0.006, was observed in OA samples. Conversely, in HC samples, heightened infiltration was noted for NK cells activated, *p* = 0.004, mast cells resting, *p* = 0.005, T cells CD4 memory resting, *p* < 0.001, and mast cells activated, *p* = 0.001. Here, we believe that the cause of the increased mast cell infiltration in the HC group may involve: (1) a specific subgroup of mast cells, and (2) factors such as the age and weight of the HC samples selected in the GEO dataset. Lastly, we conducted a correlation analysis between the expression of core genes and the infiltration of the 22 immune cell types across all samples, as depicted in Figure 6C.

### 3.7 Identification of genes that are specific to the disease

**Figure 7:**
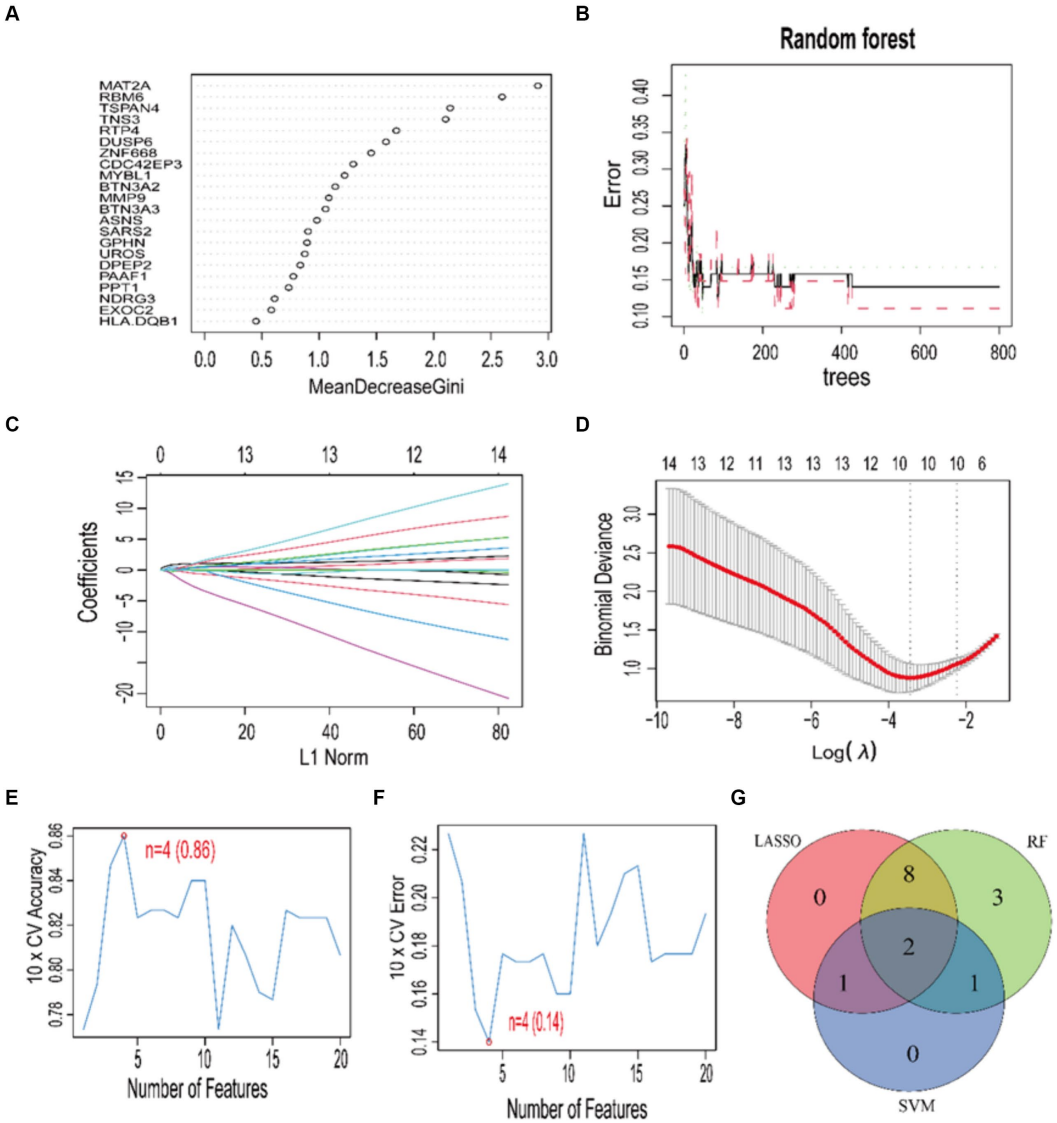
Feature gene selection using RF, LASSO, SVM algorithms. **(A)** The RF gene significance ranking. **(B)** The connection between the error rate in RF and the quantity of trees. **(C,D)** Coefficient plot and LASSO model for tenfold cross-validation. The ideal lambda value is shown by the vertical dashed line. Plots of accuracy and cross-validation error for SVM are shown in **(E,F)**. Venn diagram **(G)** showing the outcomes of the three algorithms.

Using three machine learning algorithms, specifically RF, LASSO, and SVM, we performed a secondary selection on the set of 22 core genes. Employing the RF algorithm, we discerned 14 feature genes with a relative importance exceeding 0.9 (Figures 7A,B), which encompassed: MAT2A, RBM6, TSPAN4, TNS3, RTP4, DUSP6, ZNF668, CDC42EP3, MYBL1, BTN3A2, MMP9, BTN3A3, ASNS, SARS2. Applying the LASSO algorithm, we determined the number of genes corresponding to the minimum cross-validation error (Figures 7C,D), resulting in the identification of a total of 11 feature genes: TSPAN4, TNS3, ZNF668, RBM6, CDC42EP3, MMP9, MYBL1, SARS2, PAAF1, BTN3A2, MAT2A. By utilizing the SVM algorithm, we determined the number of genes corresponding to the minimum cross-validation error (Figures 7E,F), resulting in the identification of a total of 4 feature genes: RBM6, RTP4, PAAF1, MAT2A. Following this, we performed a cross-analysis of the feature genes derived from the three algorithms (Figure 7G), leading to the identification of two disease diagnostic signature genes: MAT2A and RBM6.

### 3.8 The causal relationship between RBM6, MAT2A, and OA

**Figure 8:**
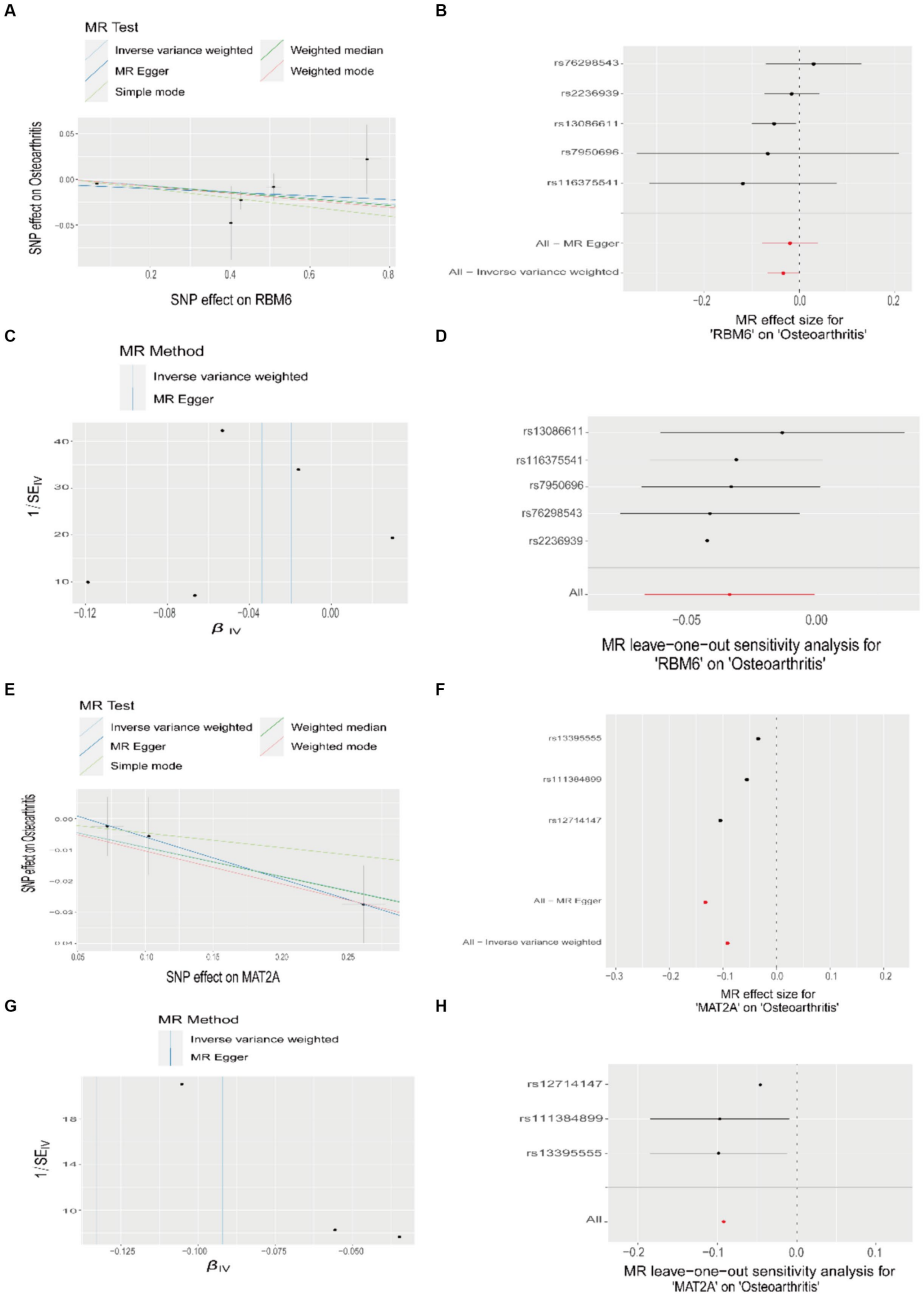
Mendelian randomization study results on the relationship between RBM6, MAT2A, and OA. **(A,E)** Scatter plots illustrating the causal relationship between RBM6, MAT2A, and OA. **(B,F)** Forest plots depicting the causal relationship of each SNP with OA risk. **(C,G)** Funnel plots assessing the reliability of the causal relationship between RBM6, MAT2A, and OA. **(D,H)** Visualization of the causal effects of RBM6, MAT2A on OA risk when omitting one SNP at a time.

We visualized the causative link between RBM6, MAT2A, and OA (Figures 8A,E), building upon the findings of the Mendelian randomization study. Using the IVW method, we revealed the correlation between the risk of OA and the expression of these two disease diagnostic genes. In this analysis, the odds ratio for RBM6 was 0.967, 95% CI = 0.935–0.999, *p* = 0.046, and for MAT2A, the odds ratio was 0.912, 95% CI = 0.840–0.990, *p* = 0.028. Simultaneously, we assessed the impact of each SNP on the outcome of OA (Figures 8B,F). Correspondingly, from top to bottom, the funnel plot shape also exhibited approximate symmetry, and no horizontal pleiotropy was observed in the MR Egger regression intercept (Figures 8C,G). Finally, we conducted MR analysis again by sequentially removing each SNP, and the corresponding results remained consistent with the previous findings (Figures 8D,H). This suggests that the calculated outcomes of all SNPs make substantial contributions to the ultimate causal relationship, avoiding dominance by any specific SNP.

### 3.9 Establishment and evaluation of the clinical prediction model

**Figure 9:**
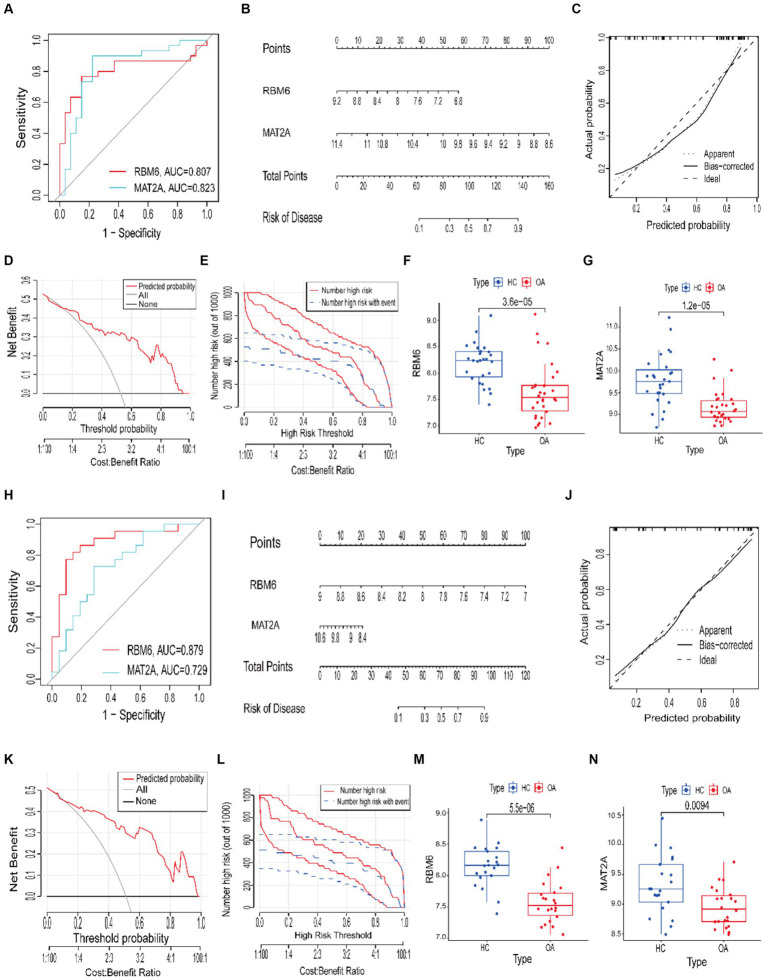
Construction of ROC curves and nomogram clinical prognosis models. **(A,H)** The training and validation sets’ ROC curve models. **(B,I)** Nomogram risk prognosis models. **(C,J)** Calibration curves. **(D,K)** Decision curve analysis (DCA) curves in the training and validation sets. **(E,L)** Clinical impact curve. **(F,G)** Boxplots depicting differential analysis of RBM6 and MAT2A in the training set samples. **(M,N)** Boxplots depicting differential analysis of RBM6 and MAT2A in the validation set samples.

To evaluate the specificity and sensitivity of RBM6 and MAT2A in diagnosing OA, ROC curves and the accompanying AUC values were employed. A model demonstrating an AUC value exceeding 0.7 is indicative of a robust predictive performance. In the training set samples, RBM6 and MAT2A, the two diagnostic marker genes, had ROC curve AUC values of 0.807 and 0.823, respectively (Figure 9A). Correspondingly, in the validation set samples, RBM6 and MAT2A, the two diagnostic marker genes, had ROC curve AUC values of 0.879 and 0.729, respectively (Figure 9H).

Subsequently, based upon sample data from the training and validation sets, we constructed a nomogram clinical prognosis model for RBM6 and MAT2A to assess their clinical diagnostic value for OA (Figures 9B,I). Each marker gene in the nomogram is assigned a score, and the overall score is obtained by summing the scores of all marker genes, corresponding to different risk levels for OA. Clearly, both in the samples from the training set and validation set, the predictive accuracy of RBM6 and MAT2A for the likelihood of OA surpasses 90%. Furthermore, to evaluate the model’s accuracy (Figures 9C,J), calibration curves were employed., where a slope closer to 1 signifies higher accuracy. DCA and clinical impact curve were utilized to assess the predictive performance of the model. In the former, a net benefit value greater than 0 signifies good predictive performance (Figures 9D,K). In the latter, the judgment is based on the alignment of the red solid line with the blue dashed line (Figures 9E,L). The results show that whether the model exhibits good accuracy and predictive performance in the training set samples or the validation set samples, both.

Additionally, we validated the differential expression of RBM6 and MAT2A in the samples. From the boxplots, it is evident that the expression of RBM6 (*p* = 3.6 × 10^−5^) and MAT2A (*p* = 1.2 × 10^−5^) is significantly elevated in the training set HC samples and significantly decreased in the OA samples (Figures 9F,G). Correspondingly, RBM6 (*p* = 5.5 × 10^−5^) and MAT2A (*p* = 0.0094) show significantly elevated expression in the validation set HC samples and significantly decreased expression in the OA samples (Figures 9M,N). The consistency of this result aligns with the conclusions derived from the Mendelian randomization analysis.

### 3.10 The expression of RBM6 and MAT2A in synovial tissue

**Figure 10:**
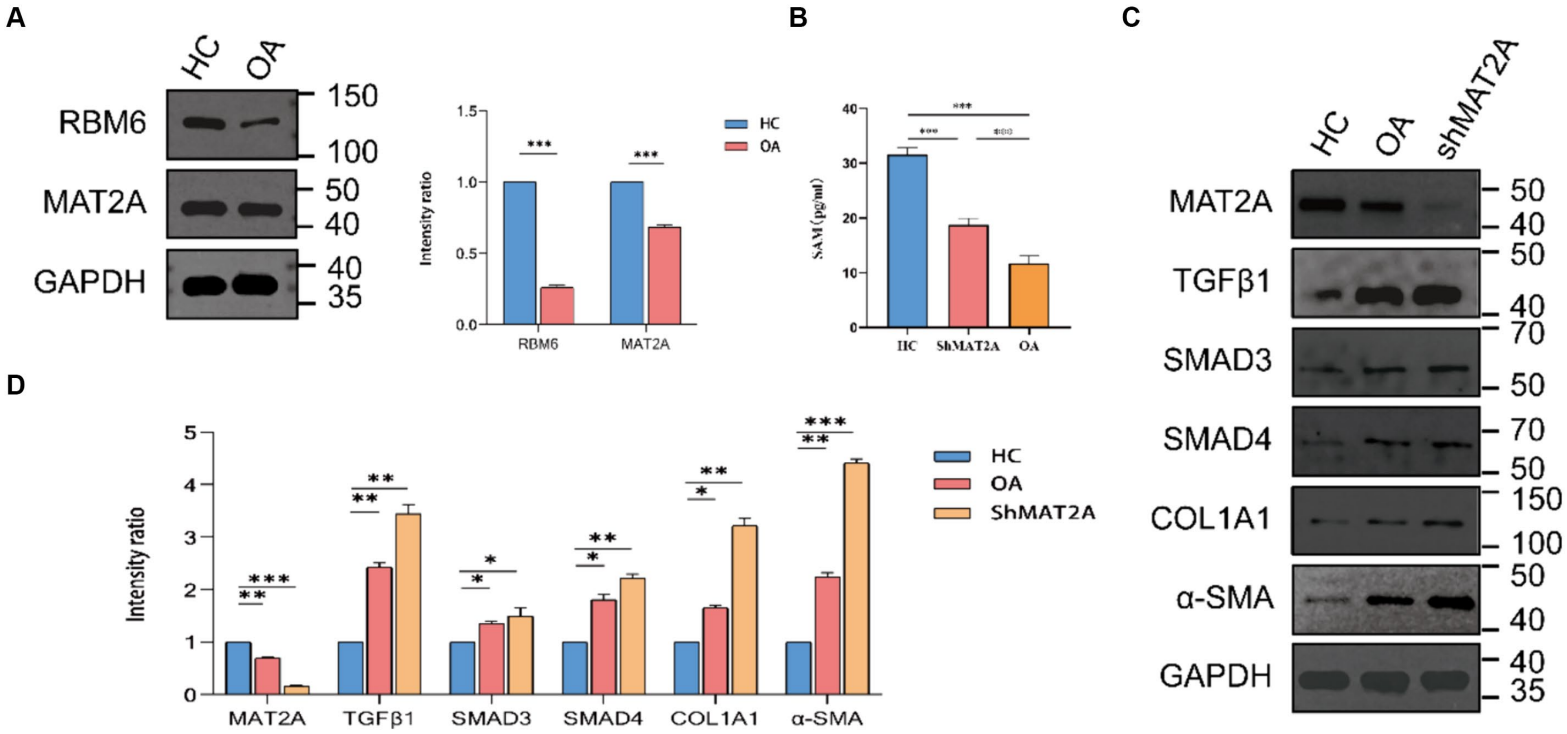
Expression of RBM6 and MAT2A in synovial cells. **(A)** Western blot analysis and quantitative bar graph of RBM6 and MAT2A. **(B)** ELISA assay determining the expression levels of SAM in the HC group, ShMAT2A group, and OA group. **(C,D)** Western blot analysis and quantitative bar graph of the expression of proteins in the TGF-β1 pathway in each group.

We performed an initial validation of the expression of diagnostic markers, namely RBM6 and MAT2A, in synovial tissues obtained from both HC and individuals with OA through Western blot analysis (Figure 10A). The corresponding results corroborate the findings of previous analyses, showing elevated expression of RBM6 and MAT2A in synovial tissue from HC, while demonstrating reduced expression in synovial tissue from OA subjects.

Building upon previous research findings, we further investigate the association between MAT2A and the onset of OA. MAT2A represents a pivotal gene encoding methionine adenosyltransferase (MAT), crucially involved in regulating the biosynthesis of S-adenosylmethionine (SAM) (21). SAM serves as a primary methyl donor and precursor of glutathione (GSH). Maintaining normal levels of SAM in the liver is essential for liver health and preventing fibrosis (22). Likewise, in synovial cells, does MAT2A exert a similar influence by regulating the expression levels of SAM? Therefore, we chose to downregulate MAT2A expression in synovial cells obtained from HC and evaluated the respective SAM levels in each group employing the ELISA method (Figure 10B). The results indicate a noteworthy decrease in SAM expression levels within synovial cells from both the ShMAT2A group and the OA group, in comparison to the HC group. This suggests that MAT2A plays a similar role in regulating SAM expression within synovial cells.

### 3.11 SAM inhibits synovial fibrosis induced by the TGF-β1-stimulated Smad3/4 pathway

Based on an abundance of prior research, it is clear that SAM demonstrates notable therapeutic efficacy in OA. In a randomized double-blind clinical trial, Bradley et al. (23) treated 81 patients with OA using SAM, leading to a noteworthy improvement in the mobility of the participants Coincidentally, Kim et al. (24) showcased the effectiveness of SAM in relieving joint pain among patients with OA in an 8-week multicenter, randomized, double-blind, placebo-controlled phase IV clinical trial. Moreover, in a recent study conducted within the past year, SAM has been confirmed to demonstrate comparable therapeutic efficacy in the treatment of OA, when compared to traditional medications like glucosamine sulfate (GS) and non-animal sourced chondroitin sulfate (naCS) (25). The aforementioned studies provide additional support to the assertion that maintaining optimal expression levels of SAM is crucial for preventing OA.

The TGF-β1 pathway is the most common pathway implicated in inducing synovial fibrosis. Upon activation of TGF-β1, it engages with its receptor, facilitating the assembly and activation of the SMAD3/4 complex, leading to the subsequent expression of fibrosis-related genes (α-SMA, COL1A1) downstream (26, 27). In this context, we hypothesize that a key factor contributing to joint osteoarthritis-like changes could be the diminished secretion of SAM in the synovium, leading to the activation of the TGF-β1-stimulated Smad3/4 signaling pathway. Western blot analysis (Figures 10C,D) was utilized for the preliminary validation of TGF-β1, SMAD3, SMAD4, α-SMA, and COL1A1 expression in the OA group, ShMAT2A group, and HC group. Results reveal a significant upregulation in the TGF-β1 pathway and its downstream fibrotic markers in synovial cells from both the ShMAT2A group and the OA group, compared to the HC group.

## 4 Discussion

Osteoarthritis primarily occurs in the elderly population, emerging as the most prevalent chronic, degenerative joint disorder clinically linked to pain and disability (28). It is not limited to affecting a single tissue; instead, it involves pathological changes throughout the entire joint. Among these, persistent low-grade synovial inflammation is considered the initiating and end point precipitating irreversible joint damage (29, 30). Nevertheless, a conclusive consensus regarding the exact pathogenesis of OA continues to be elusive. This has also resulted in a lack of effective therapeutic agents and modalities, concurrently placing a significant economic burden on both the healthcare system and the households of affected individuals. However, more lamentably, OA is a silent malady before the onset of typical clinical symptoms and radiographic alterations (5, 6). Consequently, the exploration of highly sensitive and efficient diagnostic biomarkers for OA, along with the investigation of their associated mechanistic pathways, has emerged as the prevailing focus of current research endeavors. This enables a more precise prediction of the patient’s condition progression, facilitating the delivery of personalized diagnostic and therapeutic interventions.

In this endeavor, we initially integrated summary-level data from GWAS on OA with eQTL data to explore genes associated with expression levels and complex traits. Following Mendelian randomization analysis and subsequent meticulous filtering of the corresponding outcomes, we ultimately identified 318 genes causally linked to the occurrence and progression of OA, comprising 173 low-risk genes and 145 high-risk genes. High/low-risk genes refer to genes where an elevation in gene expression correlates with an augmented or diminished likelihood of OA occurrence, respectively. Subsequently, we integrated data samples from the training set of the GEO database, which included synovial samples from both HC and individuals with OA, for conducting differential analysis. Two thousand two hundred fifty-eight DEGs were found, with 978 genes showing downregulation and 1,280 genes showing upregulation. Simultaneously, to unravel the associations between genes and traits, we conducted WGCNA on the training set data samples, acquiring the pertinent key module genes. Subsequently, through the cross-referencing of genes from the aforementioned results, we ultimately delineated the core genes exhibiting a significant causal relationship with OA traits. These core genes can be categorized into 17 low-risk genes and 5 high-risk genes. We presented the Mendelian randomization results of these 22 core genes, along with their analysis of interrelatedness, using forest plots and circle plots. This provided additional insight into the causal relationship between gene expression and disease, while investigating the functional and interaction mechanisms among different core genes. Among these, the IVW method indicated an OR of 0.967 with a 95% CI of 0.935–0.999 and *p* = 0.046 for RBM6, and an OR of 0.912 with a 95% CI of 0.840–0.990 and *p* = 0.028 for MAT2A. RBM6 showed substantial positive correlations with the expression of MYBL1, MAT2A, DUSP6, and MMP9, and a negative correlation with the expression of GPHN. MAT2A displayed marked negative correlations with the expression of BTN3A2, SARS2, and UROS, while exhibiting a positive correlation with the expression of MYBL1. To further elucidate the biological functions associated with these core genes, we performed GO and KEGG enrichment analyses. The pertinent results indicate that these core genes are predominantly involved in the T-cell receptor signaling pathway, regulating immune responses. Simultaneously, they exhibit significant enrichment in pathways related to sulfur compound biosynthesis, metabolic processes, and participate in pathways associated with amino acid biosynthesis. Among these, MAT2A is particularly enriched in the metabolic process of SAM. Moreover, considering OA as an inflammatory disease, we explored the association between immune cell infiltration in OA and the expression of core genes. A notable escalation in the infiltration levels of plasma cells, *p* = 0.001, and macrophages M0, *p* = 0.006, was observed in OA samples. Conversely, in HC samples, heightened infiltration was noted for NK cells activated, *p* = 0.004, mast cells resting, *p* = 0.005, T cells CD4 memory resting, *p* < 0.001, and mast cells activated, *p* = 0.001. Among these, the expression of RBM6 is negatively correlated with activated NK cells, *R* = 0.51, *p* = 0.004, and resting mast cells, *R* = 0.66, *p* = 0.0001, while positively correlated with macrophages M0, *R* = 0.40, *p* = 0.02, and activated mast cells, *R* = 0.48, *p* = 0.006. The expression of MAT2A is negatively correlated with mast cells resting (*R* = 0.55, *p* = 0.001) and positively correlated with mast cells activated (*R* = 0.58, *p* = 0.0007) and macrophages M0 (*R* = 0.47, *p* = 0.008). This suggests the involvement of RBM6 and MAT2A in the inflammatory response process of OA, potentially linked to the body’s self-protective mechanisms during the initial stages of the disease. To augment the representativeness and clinical utility of the screened genes, we utilized three machine learning algorithms—RF, LASSO, and SVM—to reevaluate the 22 core genes. Upon cross-referencing the genes from the respective results, we ultimately identified two diagnostic marker genes: RBM6 and MAT2A (31). Subsequently, we constructed ROC curves and nomogram clinical prediction models for RBM6 and MAT2A, with the goal of assessing their clinical diagnostic and prognostic significance for OA. The results reveal that in the training set samples, the AUC values for the ROC curves of RBM6 and MAT2A are 0.807 and 0.823, respectively. Correspondingly, in the validation set samples, the AUC values for the ROC curves of RBM6 and MAT2A are 0.879 and 0.729, respectively. In the nomogram, for both the training set and validation set samples, the prediction of the likelihood of OA by RBM6 and MAT2A surpasses 90%. The respective clinical calibration curve slopes closely approach 1, and the net benefit values on the decision curve are consistently greater than 0. This strongly supports the superior diagnostic capabilities and predictive efficacy of both models for OA.

MAT2A represents a pivotal gene encoding MAT, crucially involved in regulating the biosynthesis of SAM (21). SAM serves as a primary methyl donor and precursor of GSH. Maintaining normal levels of SAM in the liver is essential for liver health and preventing fibrosis (22). Likewise, in synovial cells, does MAT2A exert a similar influence by regulating the expression levels of SAM? Building upon previous research findings, we chose to downregulate MAT2A expression in synovial cells obtained from HC and evaluated the respective SAM levels in each group employing the ELISA method. The results indicate a noteworthy decrease in SAM expression levels within synovial cells from both the ShMAT2A group and the OA group, in comparison to the HC group. This suggests that MAT2A plays a similar role in regulating SAM expression within synovial cells. The TGF-β1 pathway is the most common pathway implicated in inducing synovial fibrosis. Upon activation of TGF-β1, it engages with its receptor, facilitating the assembly and activation of the SMAD3/4 complex, leading to the subsequent expression of fibrosis-related genes (α-SMA, COL1A1) downstream (26, 27). Based on an abundance of prior research, it is clear that SAM demonstrates notable therapeutic efficacy in OA (23–25). In this context, we hypothesize that a key factor contributing to joint osteoarthritis-like changes could be the diminished secretion of SAM in the synovium, leading to the activation of the TGF-β1-stimulated Smad3/4 signaling pathway. Thus, we employed western blot analysis to preliminarily validate the expression of TGF-β1, SMAD3, SMAD4, α-SMA, and COL1A1 in the HC group, ShMAT2A group, and OA group. The results show a noteworthy increase in the expression levels of the TGF-β1 pathway and its downstream fibrotic markers in synovial cells from both the ShMAT2A group and the OA group, when compared to the HC group. Nevertheless, our study is subject to certain limitations. Owing to various constraints, we were only able to conduct preliminary validation of the relevant hypotheses based on previous research. Moreover, the limited number of relevant samples may introduce a degree of bias.

## 5 Conclusion

This work offers the first proof that RBM6 and MAT2A serve as robust diagnostic indicators for OA. MAT2A, through its involvement in regulating the synthesis of SAM, inhibits the activation of the TGF-β1-induced Smad3/4 signaling pathway, thereby effectively averting the possibility of synovial fibrosis. Concurrently, the development of a prognostic risk model facilitates early OA diagnosis, functional recovery evaluation, and offers direction for further therapy.

## Data availability statement

This data can be found here: publicly available datasets were analyzed in this study. This data can be found here: the datasets generated and/or analyzed in this study can be accessed at the GWAS database (https://gwas.mrcieu.ac.uk/), eQTLGen consortium (https://www.eqtlgen.org/), and GEO database (https://www.ncbi.nlm.nih.gov/geo).

## Ethics statement

The studies involving humans were approved by the clinical specimen collection plan for synovial tissues from both HC and OA has received approval from the Ethics Committee of the Second Affiliated Hospital of Anhui Medical University (Approval Number: YX2022-104). The studies were conducted in accordance with the local legislation and institutional requirements. The participants provided their written informed consent to participate in this study.

## Author contributions

YY: Writing – review & editing, Writing – original draft, Visualization, Validation, Software, Methodology, Investigation, Formal analysis, Data curation, Conceptualization. JH: Writing – review & editing, Data curation. ZX: Writing – review & editing, Data curation. CW: Writing – review & editing, Data curation. ZH: Writing – review & editing, Data curation. CZ: Writing – review & editing, Resources, Funding acquisition, Data curation. WC: Writing – review & editing, Supervision, Resources, Project administration, Methodology, Funding acquisition, Data curation.
