# Supplementary material for: Mendelian randomization based on genome-wide association studies and expression quantitative trait loci, predicting gene targets for the complexity of osteoarthritis as well as the clinical prognosis of the condition
Source: Front Med (Lausanne). 2024 Jun 26;11:1409439. doi: 10.3389/fmed.2024.1409439 (PMC11238174; doi:10.3389/fmed.2024.1409439)
Supplement: Supplementary file 1 [file Data_Sheet_1.docx]

**STROBE-MR checklist of recommended items to address in reports of Mendelian randomization studies**^1^ ^2^

| **Item No.** | **Section** | **Checklist item** | **Page No.** | **Relevant text from manuscript** |
| --- | --- | --- | --- | --- |
| 1 | **TITLE and ABSTRACT** | Indicate Mendelian randomization (MR) as the study’s design in the title and/or the abstract if that is a main purpose of the study | 2 | Mendelian Randomization based on GWAS and eQTL, predicting gene targets for the complexity of osteoarthritis as well as the clinical prognosis of the condition. |
|  | **INTRODUCTION** |  | 2 |  |
| 2 | **Background** | Explain the scientific background and rationale for the reported study. What is the exposure? Is a potential causal relationship between exposure and outcome plausible? Justify why MR is a helpful method to address the study question | 2 | Osteoarthritis (OA) entails a prevalent chronic ailment, marked by the widespread involvement of entire joints. Prolonged low-grade synovial inflammation serves as the key instigator for a cascade of pathological alterations in the joints. We believe that synovial inflammation alters the production of certain substances, thereby triggering OA, and this process is closely associated with peripheral blood mononuclear cells. Therefore, we selected 31,684 blood and peripheral blood mononuclear cell samples from the eQTLGen Consortium as exposure data, screening out SNPs with cis-eQTL P values < 5 × 10^-8 as IVs. The potential causal relationship between exposure and outcome is credible. Our study is based on Mendelian randomization, but it is just one of the main threads in our research. We have employed different methods to confirm the robustness of our findings. |
| 3 | **Objectives** | State specific objectives clearly, including pre-specified causal hypotheses (if any). State that MR is a method that, under specific assumptions, intends to estimate causal effects | 2 | We believe that synovial inflammation alters the production of certain substances, thereby triggering OA, and this process is closely associated with peripheral blood mononuclear cells. |
|  | **METHODS** |  |  |  |
| 4 | **Study design and data sources** | Present key elements of the study design early in the article. Consider including a table listing sources of data for all phases of the study. For each data source contributing to the analysis, describe the following: | 4 | The Mendelian randomization study is just one component of our research, and the data for this part are sourced entirely from public databases, with detailed dataset IDs provided in the article. The datasets generated and/or analyzed in this study can be accessed at the GWAS database (https://gwas.mrcieu.ac.uk/), eQTLGen Consortium (https://www.eqtlgen.org/), and GEO database (https://www.ncbi.nlm.nih.gov/geo). |
|  | a) | Setting: Describe the study design and the underlying population, if possible. Describe the setting, locations, and relevant dates, including periods of recruitment, exposure, follow-up, and data collection, when available. | 4 | The data utilized in our study are sourced from the GWAS database and the eQTLGen Consortium, with the dataset ID for knee osteoarthritis being ebi-a-GCST007090. |
|  | b) | Participants: Give the eligibility criteria, and the sources and methods of selection of participants. Report the sample size, and whether any power or sample size calculations were carried out prior to the main analysis | 4 | NA |
|  | c) | Describe measurement, quality control and selection of genetic variants | 4 | NA |
|  | d) | For each exposure, outcome, and other relevant variables, describe methods of assessment and diagnostic criteria for diseases | 4 | Assessed through five methods, namely MR Egger, inverse variance weighted (IVW), weighted median, weighted mode and simple mode, utilizing the "TwoSampleMR" package within the R software |
|  | e) | Provide details of ethics committee approval and participant informed consent, if relevant |  | The data for Mendelian randomization research are sourced from public databases, and detailed dataset IDs are provided in the article. Hence, this section does not require ethical approval or patient informed consent. |
| 5 | **Assumptions** | Explicitly state the three core IV assumptions for the main analysis (relevance, independence and exclusion restriction) as well assumptions for any additional or sensitivity analysis | 4 | Select appropriate SNPs as IVs to explore causal effects between exposure data and outcome data. The eligible IVs must satisfy the following three conditions: (1) Correlation Assumption: SNPs exhibit close associations with the exposure. (2) Independence Assumption: IVs maintain independence from confounding factors. (3) Exclusion Assumption: There is no direct association between IVs and the outcome. The specific screening methodology has been delineated in the preceding text. |
| 6 | **Statistical methods: main analysis** | Describe statistical methods and statistics used | 4 | We conducted statistical analysis using the TwoSampleMR R software package. |
|  | a) | Describe how quantitative variables were handled in the analyses (i.e., scale, units, model) | 4 | NA |
|  | b) | Describe how genetic variants were handled in the analyses and, if applicable, how their weights were selected | 4 | NA |
|  | c) | Describe the MR estimator (e.g. two-stage least squares, Wald ratio) and related statistics. Detail the included covariates and, in case of two-sample MR, whether the same covariate set was used for adjustment in the two samples | 4 | We adjusted using the same set of confounding factors to ensure consistency and comparability of results. |
|  | d) | Explain how missing data were addressed | 4 | We utilized the appropriate R packages and conducted sensitivity analysis. |
|  | e) | If applicable, indicate how multiple testing was addressed | 4 | We utilized the appropriate R packages, The article has elaborated on this in detail. |
| 7 | **Assessment of assumptions** | Describe any methods or prior knowledge used to assess the assumptions or justify their validity | 4 | Assessed through five methods, namely MR Egger, inverse variance weighted (IVW), weighted median, weighted mode and simple mode, utilizing the "TwoSampleMR" package within the R software,  Additionally, we further validated the credibility of the results by employing various machine learning methods and constructing relevant clinical prognosis models. |
| 8 | **Sensitivity analyses and additional analyses** | Describe any sensitivity analyses or additional analyses performed (e.g. comparison of effect estimates from different approaches, independent replication, bias analytic techniques, validation of instruments, simulations) | 4 | We utilized the appropriate R packages |
| 9 | **Software and pre-registration** |  |  |  |
|  | a) | Name statistical software and package(s), including version and settings used |  | R 4.3.2  The relevant screening criteria have been elucidated in the article. |
|  | b) | State whether the study protocol and details were pre-registered (as well as when and where) |  | NA |
|  | **RESULTS** |  |  |  |
| 10 | **Descriptive data** |  |  | As this study is not solely a Mendelian randomization analysis, the following does not apply. We conducted all analyses using the appropriate R packages, and all data are sourced from public databases, with relevant details presented in the article. If needed, we can provide the analyzed data in the supplementary materials. |
|  | a) | Report the numbers of individuals at each stage of included studies and reasons for exclusion. Consider use of a flow diagram |  |  |
|  | b) | Report summary statistics for phenotypic exposure(s), outcome(s), and other relevant variables (e.g. means, SDs, proportions) |  | NA |
|  | c) | If the data sources include meta-analyses of previous studies, provide the assessments of heterogeneity across these studies |  | NA |
|  | d) | For two-sample MR:  i.  Provide justification of the similarity of the genetic variant-exposure associations between the exposure and outcome samples  ii.  Provide information on the number of individuals who overlap between the exposure and outcome studies |  | NA |
| 11 | **Main results** |  |  | We visualized the results using odds ratios and related scatterplot forest plots. |
|  | a) | Report the associations between genetic variant and exposure, and between genetic variant and outcome, preferably on an interpretable scale | 8 | Used |
|  | b) | Report MR estimates of the relationship between exposure and outcome, and the measures of uncertainty from the MR analysis, on an interpretable scale, such as odds ratio or relative risk per SD difference | 8 | Used |
|  | c) | If relevant, consider translating estimates of relative risk into absolute risk for a meaningful time period | 8 | Used |
|  | d) | Consider plots to visualize results (e.g. forest plot, scatterplot of associations between genetic variants and outcome versus between genetic variants and exposure) | 10 | Used |
| 12 | **Assessment of assumptions** |  |  | We conducted sensitivity analysis and multiplicity analysis, and if needed, these can be uploaded as supplementary materials. |
|  | a) | Report the assessment of the validity of the assumptions | 8 | Used |
|  | b) | Report any additional statistics (e.g., assessments of heterogeneity across genetic variants, such as *I^2^*, Q statistic or E-value) | 10 | Used |
| 13 | **Sensitivity analyses and additional analyses** |  |  |  |
|  | a) | Report any sensitivity analyses to assess the robustness of the main results to violations of the assumptions | 10 | Used |
|  | b) | Report results from other sensitivity analyses or additional analyses | 10 | Used |
|  | c) | Report any assessment of direction of causal relationship (e.g., bidirectional MR) | 10 | Used |
|  | d) | When relevant, report and compare with estimates from non-MR analyses | 10 | Used |
|  | e) | Consider additional plots to visualize results (e.g., leave-one-out analyses) | 10 | Used |
|  | **DISCUSSION** |  |  |  |
| 14 | **Key results** | Summarize key results with reference to study objectives | 10 | We visualized the causative link between RBM6, MAT2A, and OA |
| 15 | **Limitations** | Discuss limitations of the study, taking into account the validity of the IV assumptions, other sources of potential bias, and imprecision. Discuss both direction and magnitude of any potential bias and any efforts to address them |  | As this study is not solely a Mendelian randomization analysis, the following does not apply. |
| 16 | **Interpretation** |  |  | As this study is not solely a Mendelian randomization analysis, the following does not apply |
|  | a) | Meaning: Give a cautious overall interpretation of results in the context of their limitations and in comparison with other studies |  | NA |
|  | b) | Mechanism: Discuss underlying biological mechanisms that could drive a potential causal relationship between the investigated exposure and the outcome, and whether the gene-environment equivalence assumption is reasonable. Use causal language carefully, clarifying that IV estimates may provide causal effects only under certain assumptions |  | NA |
|  | c) | Clinical relevance: Discuss whether the results have clinical or public policy relevance, and to what extent they inform effect sizes of possible interventions |  | NA |
| 17 | **Generalizability** | Discuss the generalizability of the study results (a) to other populations, (b) across other exposure periods/timings, and (c) across other levels of exposure |  | applicability |
|  | **OTHER INFORMATION** |  |  |  |
| 18 | **Funding** | Describe sources of funding and the role of funders in the present study and, if applicable, sources of funding for the databases and original study or studies on which the present study is based |  | Already detailed in the article. |
| 19 | **Data and data sharing** | Provide the data used to perform all analyses or report where and how the data can be accessed, and reference these sources in the article. Provide the statistical code needed to reproduce the results in the article, or report whether the code is publicly accessible and if so, where |  | Already detailed in the article. |
| 20 | **Conflicts of Interest** | All authors should declare all potential conflicts of interest |  | Already detailed in the article. |

This checklist is copyrighted by the Equator Network under the Creative Commons Attribution 3.0 Unported (CC BY 3.0) license.

1. Skrivankova VW, Richmond RC, Woolf BAR, Yarmolinsky J, Davies NM, Swanson SA, et al. Strengthening the Reporting of Observational Studies in Epidemiology using Mendelian Randomization (STROBE-MR) Statement. JAMA. 2021;under review.

2. Skrivankova VW, Richmond RC, Woolf BAR, Davies NM, Swanson SA, VanderWeele TJ, et al. Strengthening the Reporting of Observational Studies in Epidemiology using Mendelian Randomisation (STROBE-MR): Explanation and Elaboration. BMJ. 2021;375:n2233.
