# Supplementary material for: Mendelian randomization based on genome-wide association studies and expression quantitative trait loci, predicting gene targets for the complexity of osteoarthritis as well as the clinical prognosis of the condition
Source: Front Med (Lausanne). 2024 Jun 26;11:1409439. doi: 10.3389/fmed.2024.1409439 (PMC11238174; doi:10.3389/fmed.2024.1409439)
Supplement: Supplementary file 2 [file Image_1.pdf]

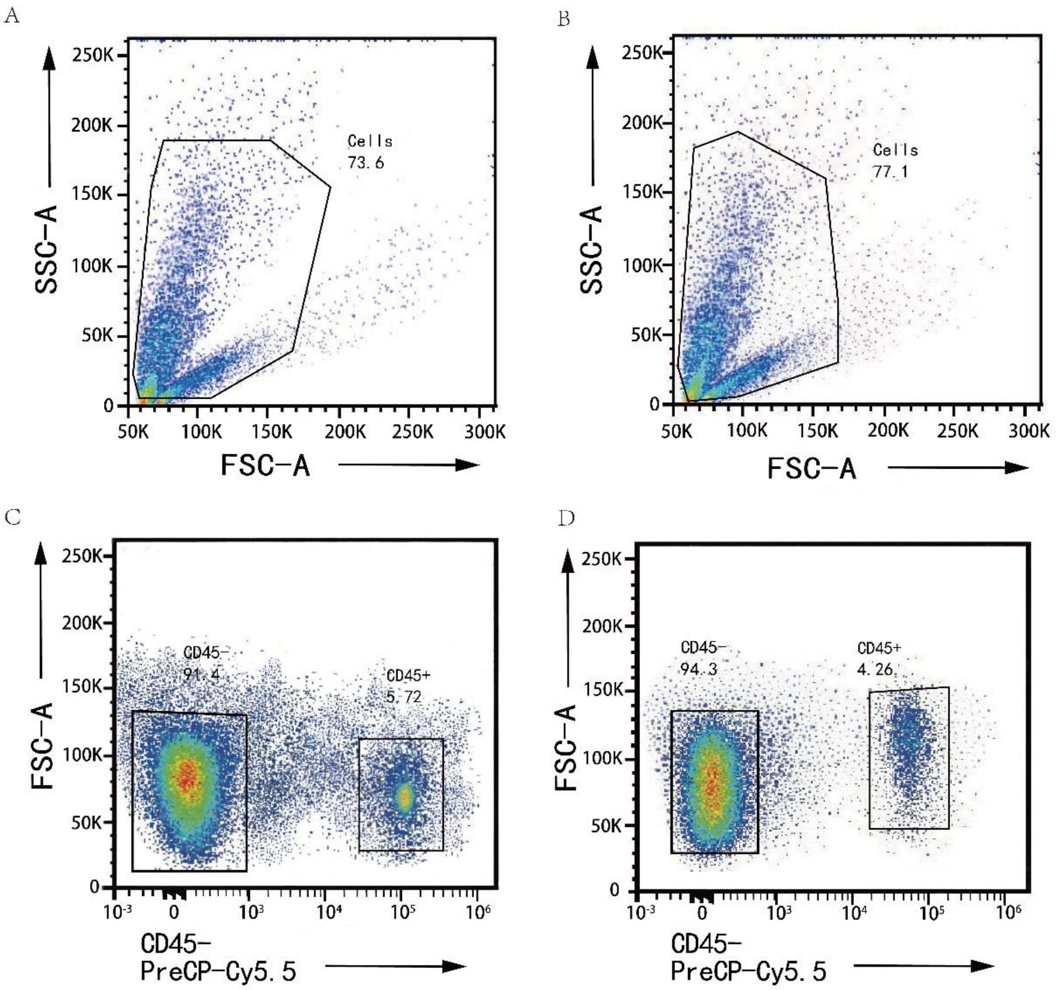

Figure S1.Flow cytometric analysis of primary synovial cells. (A, C) Primary synovial cells from healthy individuals.(B, D) Primary synovial cells from patients with OA.
