# Supplementary material for: Mendelian randomization based on genome-wide association studies and expression quantitative trait loci, predicting gene targets for the complexity of osteoarthritis as well as the clinical prognosis of the condition
Source: Front Med (Lausanne). 2024 Jun 26;11:1409439. doi: 10.3389/fmed.2024.1409439 (PMC11238174; doi:10.3389/fmed.2024.1409439)

Figure10A RBM6

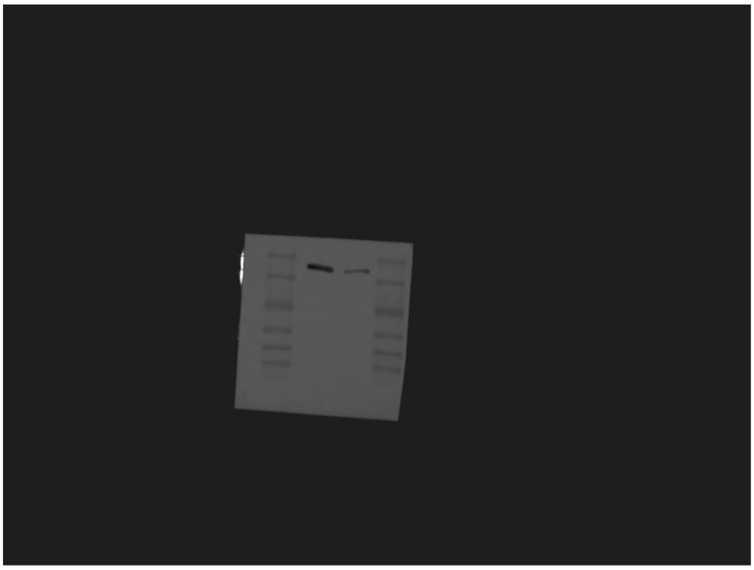

Figure10A MAT2A

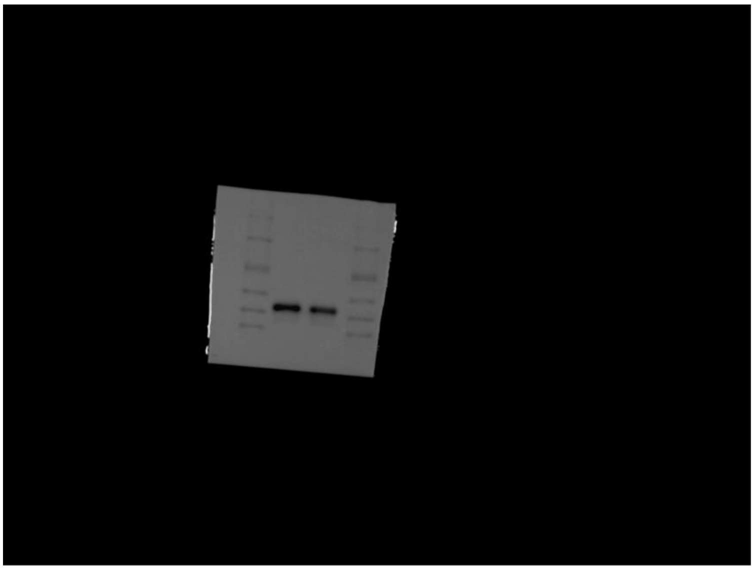

Figure10A GAPDH

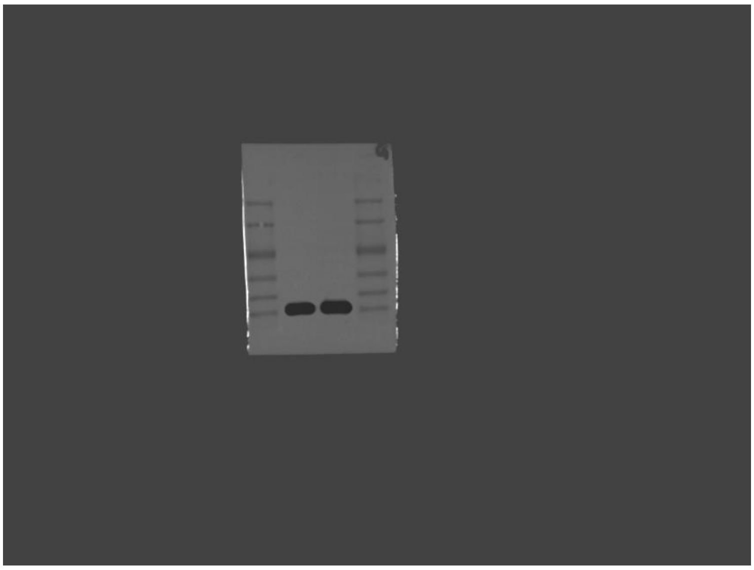

Figure10D COL1A1

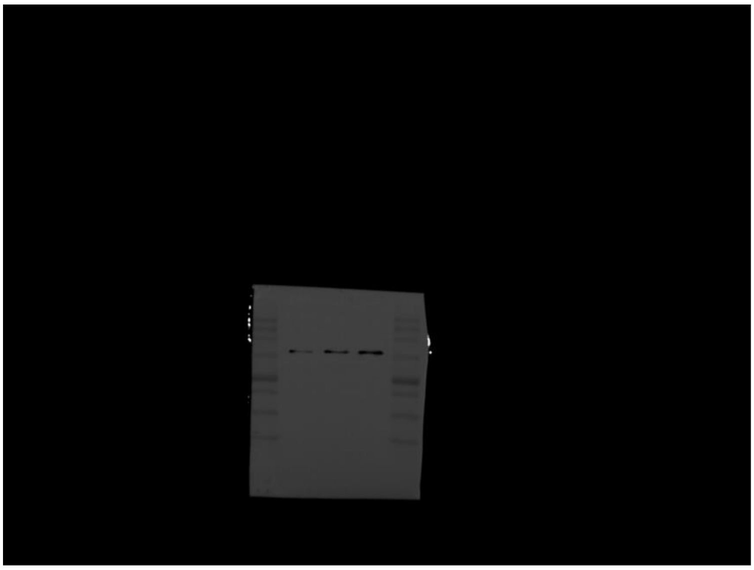

Figure10D MAT2A

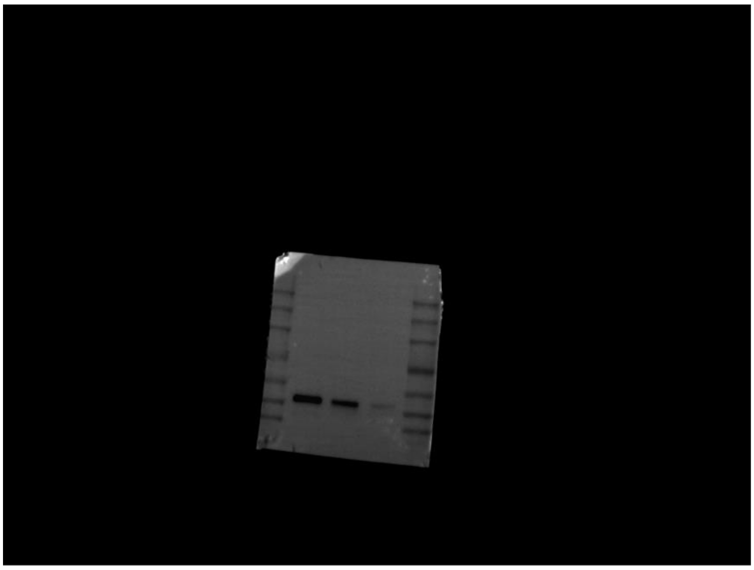

Figure10D SMAD3

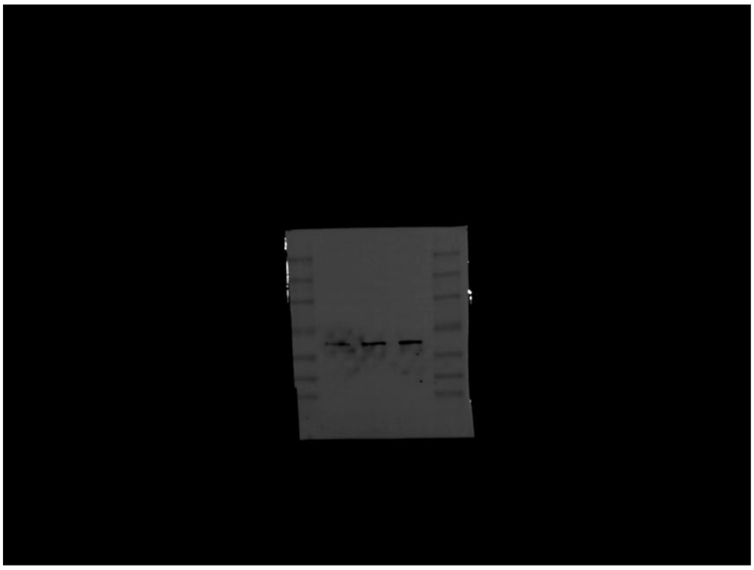

Figure10D SMAD4

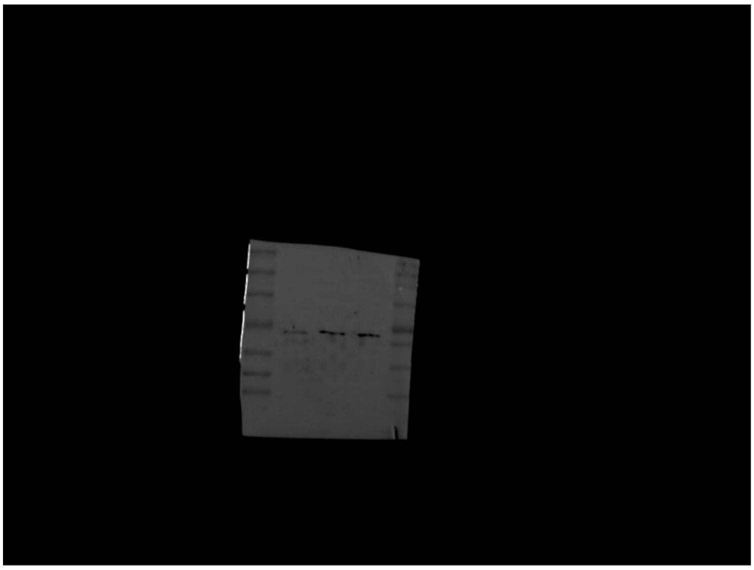

Figure10D TGFβ1

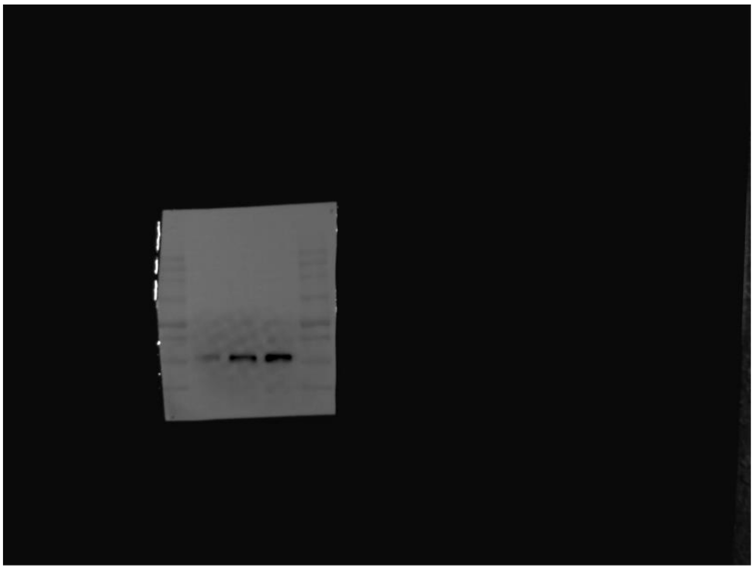

Figure10D α-SMA

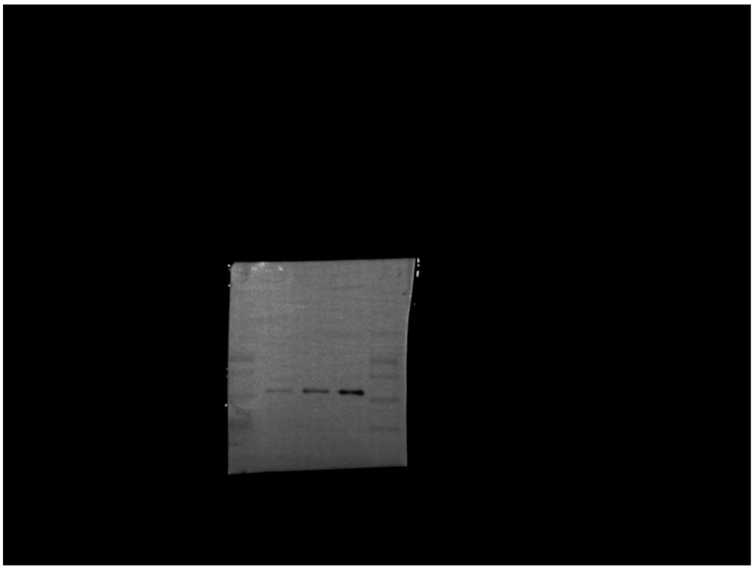

Figure10D GAPDH

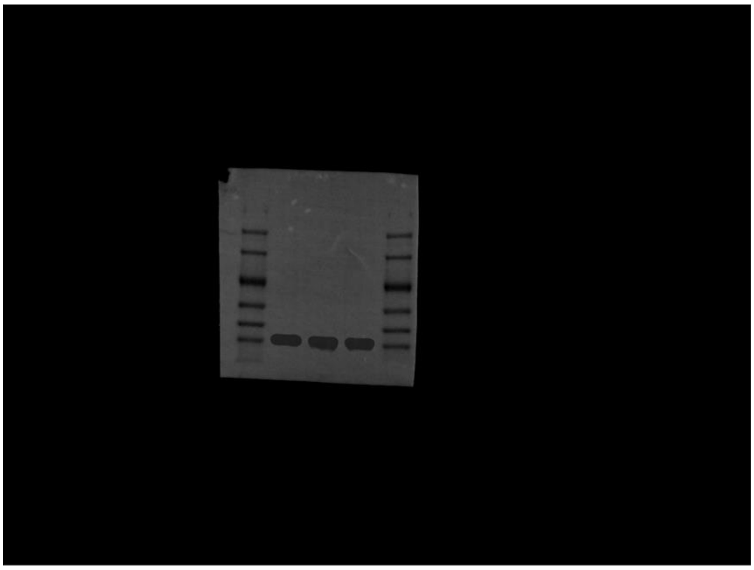

Supplement: Supplementary file 3 [file Image_2.pdf]
